# Metabolic profiling and combined therapeutic strategies unveil the cytotoxic potential of selenium-chrysin (SeChry) in NSCLC cells

**DOI:** 10.1042/BSR20240752

**Published:** 2024-07-31

**Authors:** Cindy Mendes, Isabel Lemos, Ana Hipólito, Bruna Abreu, Catarina Freitas-Dias, Filipa Martins, Rita F. Pires, Hélio Barros, Vasco D.B. Bonifácio, Luís G. Gonçalves, Jacinta Serpa

**Affiliations:** 1 iNOVA4Health, NOVA Medical School, Faculdade de Ciências Médicas, NMS, FCM, Universidade NOVA de Lisboa, Campo dos Mártires da Pátria, 130, 1169-056 Lisboa, Portugal; 2 Instituto Português de Oncologia de Lisboa Francisco Gentil (IPOLFG), Rua Prof Lima Basto 1099-023, Lisboa, Portugal; 3 IBB - Institute for Bioengineering and Biosciences, Instituto Superior Técnico, Universidade de Lisboa, Av. Rovisco Pais, 1049-001 Lisbon, Portugal; 4 Department of Bioengineering, Instituto Superior Técnico, Universidade de Lisboa, Av. Rovisco Pais, 1049-001 Lisbon, Portugal; 5 Instituto de Tecnologia Química e Biológica António Xavier (ITQB NOVA), Avenida da República (EAN), 2780-157 Oeiras, Portugal

**Keywords:** cancer metabolism, lung cancer, metabolic remodeling, new therapies, selenio-chrysin

## Abstract

Lung cancer ranks as the predominant cause of cancer-related mortalities on a global scale. Despite progress in therapeutic interventions, encompassing surgical procedures, radiation, chemotherapy, targeted therapies and immunotherapy, the overall prognosis remains unfavorable. Imbalances in redox equilibrium and disrupted redox signaling, common traits in tumors, play crucial roles in malignant progression and treatment resistance. Cancer cells, often characterized by persistent high levels of reactive oxygen species (ROS) resulting from genetic, metabolic, and microenvironmental alterations, counterbalance this by enhancing their antioxidant capacity. Cysteine availability emerges as a critical factor in chemoresistance, shaping the survival dynamics of non-small cell lung cancer (NSCLC) cells. Selenium-chrysin (SeChry) was disclosed as a modulator of cysteine intracellular availability. This study comprehensively characterizes the metabolism of SeChry and investigates its cytotoxic effects in NSCLC. SeChry treatment induces notable metabolic shifts, particularly in selenocompound metabolism, impacting crucial pathways such as glycolysis, gluconeogenesis, the tricarboxylic acid (TCA) cycle, and amino acid metabolism. Additionally, SeChry affects the levels of key metabolites such as acetate, lactate, glucose, and amino acids, contributing to disruptions in redox homeostasis and cellular biosynthesis. The combination of SeChry with other treatments, such as glycolysis inhibition and chemotherapy, results in greater efficacy. Furthermore, by exploiting NSCLC's capacity to consume lactate, the use of lactic acid-conjugated dendrimer nanoparticles for SeChry delivery is investigated, showing specificity to cancer cells expressing monocarboxylate transporters.

## Introduction

Despite advancements in prevention and novel treatment strategies, cancer remains a leading cause of premature mortality globally [1]. As reported by the International Agency for Research on Cancer (IARC) in 2020, the incidence of cancer worldwide was estimated to be between 18 and 19 million newly diagnosed cases, with a 5-year prevalence reaching approximately 44 million cases [2]. Among these, lung cancer constituted over 11% of the newly identified cases, surpassing 2.2 million instances, leading to a mortality rate of approximately 1.8 million individuals worldwide across genders [2]. The overall prognosis remains unfavorable due to the prevalent occurrence of locally advanced or metastatic tumors detected at the time of diagnosis, coupled with the presence of multiple resistance mechanisms [3].

Non-small cell lung cancer (NSCLC), constituting the predominant subgroup, makes up approximately 85% of all diagnosed cases of lung cancer [4]. The identification of driver oncogenes, such as mutations or rearrangements in the *epidermal growth factor receptor (EGFR), Kirsten rat sarcoma (KRAS), anaplastic lymphoma kinase (ALK)*, and *ROS proto-oncogene 1 (ROS1)* genes, along with the subsequent development of molecular-targeted therapies has marked the initiation of a new era in precision medicine tailored for the management of NSCLC [5]. NSCLC exhibits notable cellular heterogeneity within tumors, a characteristic that extends to the tumor microenvironment (TME) [6,7]. Moreover, akin to many other tumors, the TME of NSCLC is recognized for its abundance of diverse mediators, whose properties have been comprehensively elucidated throughout tumorigenesis [8]. The high heterogeneity observed in NSCLC poses numerous challenges in terms of prognosis and was shown to be correlated with both chemoresistance and the likelihood of metastasis [9,10].

Disease recurrence, stemming from resistance to therapies, stands as a crucial factor contributing to mortality [11]. Notably, only a limited percentage of patients, approximately 15–20%, exhibit a positive response to tyrosine kinase inhibitors (TKI) [8]. Additionally, resistance to anti-EGFR therapies typically manifests within approximately 10–13 months following treatment initiation [12,13]. Thus, it is crucial to promptly develop new approaches to address acquired resistance. Platinum-based chemotherapy continues to be a standard treatment approach for NSCLC and is commonly administered in combination with immunotherapy (immune checkpoint inhibitors) for the treatment of the majority of patients with advanced/metastatic disease [9]. Platinum drugs induce the formation of adducts of DNA and proteins, coupled with the generation of intracellular ROS, resulting in cell damage and apoptosis [14,15]. Resistance to drugs with oxidative and alkylating properties is linked to changes in the dynamics of glutathione (GSH) and cysteine, as well as alterations in the biosynthesis of hydrogen sulfide (H_2_S) catalyzed by cystathionine-β-synthase (CBS) [16,17]. Our group previously revealed the significance of cysteine availability in the context of chemoresistance, emphasizing its critical role in the survival of ovarian cancer cells [18–20]. More recently, the metabolic reliance on cysteine was also confirmed in NSCLC cell lines [21]. In NSCLC cells, despite presenting a diverse adaptive metabolic profile, the production and consumption of lactate is a defining label of some cellular subsets [22]. Lactate, besides being a crucial metabolic substrate, functions as an intercellular and inter-tissue redox signaling molecule. It serves as an energy source for oxidative metabolism in various tissues, contributing to the maintenance of redox homeostasis and overall tissue and organism integrity [23]. Intercellular lactate shuttling is facilitated by monocarboxylate transporters (MCTs), with MCT1 and MCT4 being the most extensively studied and frequently overexpressed in cancer cells, emerging as promising therapeutic targets for cancer treatment [24].

While specific genetic mutations significantly impact the clinical response to TKIs, the disruption of cellular processes, including metabolic changes, might play a role in the development of drug resistance. Interestingly, prolonged administration of gefitinib (EGFR TKI) in NSCLC cells increased ROS levels and correlated with the occurrence of epithelial–mesenchymal transition (EMT), a well-established characteristic of tumors displaying drug resistance [25]. Thus, this suggests that antioxidants could potentially provide therapeutic benefits by attenuating TKI-induced ROS and EMT [25]. The imbalance between oxidants and antioxidants determines the levels of ROS in tissues [26]. ROS-induced oxidative stress exhibits a dual role in cancer: moderate ROS levels exert positive effects, including the promotion of inflammation, tumor progression, and the development of drug resistance [27]. Conversely, an abundance of ROS can elicit negative outcomes, such as inhibiting growth or inducing the death of cancer cells [27]. EGFR-mediated tumor progression and resistance to EGFR TKIs were found to be linked to ROS-induced oxidative stress, stemming from mitochondrial dysfunction, NADPH oxidase (NOX) overactivation, and the aberrant expression of antioxidant enzymes [28,29].

We previously demonstrated that selenium-chrysin (SeChry) disrupts cysteine availability and metabolism as it induces oxidative stress, leading to GSH depletion, and inhibits CBS, thereby exerting cytotoxic effects in NSCLC [21] and ovarian cancer cells [17]. Here, we aimed to fully characterize the metabolism of SeChry and the mechanisms underlying its cytotoxicity in NSCLC. Moreover, we also tested SeChry encapsulated in polyurea (PURE) dendrimers functionalized with lactic acid (SeChry@PURE_G4_-LA_24_) as a potential therapeutic approach.

## Materials and methods

### Cell lines and culture conditions

Human adenocarcinoma cell lines A549 (CCL-185™, ATCC), H1975 (CRL-5908™, ATCC), and H522 (CRL-5810™, ATCC), adenosquamous carcinoma cell line H596 (HTB-178™, ATCC) and mucoepidermoid carcinoma cell line H292 (CRL-1848™, ATCC) were obtained from American Type Culture Collection (ATCC, Manassas, VA, U.S.A.) and adenocarcinoma cell line PC-9 (90071810, ECACC) was obtained from European Collection of Authenticated Cell Cultures (ECACC, Porton Down, Salisbury, United Kingdom). Normal human bronchial epithelium cell line BEAS-2B (CRL-3588™, ATCC) was also used. H1975, H522 and H596 were cultured in RPMI 1640 (BE12-167F, Lonza) supplemented with 0.58 g/L of L-glutamine, 10% fetal bovine serum FBS (S 0615, Merck), 1% antibiotic-antimycotic (AA) (P06-07300, PAN Biotech) and 50 μg/ml gentamicin (15750-060, Gibco, Life Technologies). The remaining cell lines were cultured in Dulbecco’s Modified Eagle’s Medium 1× (DMEM) (41965-039, Gibco, Life Technologies) supplemented with 10% FBS, 1% AA and 50 μg/ml gentamicin. Cells were maintained at 37°C in a humidified environment with 5% CO_2_. Cells were cultured until an optical confluence of 75–100% and detachment was performed with 0.05% trypsin-EDTA 1× (25300-054, Invitrogen). Before any *in vitro* experiment, cells were synchronized under starvation (FBS-free culture medium) overnight, except for cytotoxicity assays. Cells were kept in control conditions or exposed to SeChry for 24 h in the case of nuclear magnetic resonance (NMR) assays, or 48 h for cytotoxic assays. The concentrations of SeChry for each cell line were chosen according to the previously determined half-maximum effective concentrations (EC_50_) [21]. Also, 50 μM bromopyruvic acid (BPA; 16490, Sigma-Aldrich; Darmstadt, Germany) was added in cytotoxic assays for 48 h, alone or in combination with SeChry. Cisplatin and/or carboplatin (0.025 mg/ml; IPOLFG’s Pharmacy, Lisbon, Portugal) were added in the last 24 h of the 48 h cytotoxic assays. Additionally, cell lines were exposed to different concentrations (6.25–150 µM) of SeChry@PURE_G4_-LA_24_ (nanoencapsulated SeChry) for 48 h and the EC_50_ was determined. In some assays, cells were incubated overnight with MCT1 inhibitor AZD3965 (10 μM, HY-12750, MedChem Express) and/or MCT4 inhibitor AZD0095 (10 μM, HY-148517, MedChem Express) or with gefitinib (20 μM, SML1657, Sigma-Aldrich, MO, U.S.A.) before being exposed to SeChry@PURE_G4_-LA_24_. The chosen concentrations of MCT1/MCT4 inhibitors and gefitinib were based on studies previously reported in the literature [22,30,31].

### Cell death analysis

Cell death was assessed by flow cytometry. Cells (2 × 10^5^ cells/ml) were seeded in 24-well plates and exposed to conditions. After experimental conditions, supernatants (conditioned culture media) were collected, and cells were harvested with trypsin and centrifuged, together with cells in the supernatant, at 150 × ***g*** for 2 min. Cells were suspended with 0.5 μl Annexin V- fluorescein (FITC) (640906, BioLegend) in annexin V binding buffer 1 × (10 mM HEPES (pH 7.4; 391333, Millipore, New York, NY, U.S.A.), 140 mM sodium chloride (NaCl; 106404, Merck), 2.5 mM calcium chloride (CaCl_2_; 449709, SigmaAldrich) and incubated at room temperature (RT), in the dark for 15 min. After incubation, samples were resuspended in 200 μL PBS 1× 0.1% (v/w) bovine serum albumin (BSA) and centrifuged at 155 × ***g*** for 2 min. Cells were resuspended in 100 μl of annexin V binding buffer 1 × and 2.5 μl propidium iodide (PI, 50 μg/ml; P4170, Sigma-Aldrich) was added and samples were analyzed by flow cytometry (FACScalibur - Becton Dickinson), using *FlowJo* 8.7 software (https://www.flowjo.com). Experiments were performed in biological triplicates.

### Nuclear magnetic resonance (NMR) spectroscopy

Nuclear Magnetic Resonance (NMR) spectroscopy was performed on A549, H292 and PC-9 cell lines. Cells were plated in 175 cm^2^ T-Flasks: A549 (2 × 10^7^ cells/flask); H292 (1.5 × 10^7^ cells/flask) and PC-9 (2.5 × 10^7^ cells/flask), and exposed to control conditions or SeChry for 24 h. Culture media (supernatant) was collected and stored at −80 °C. Cell methanol/chloroform/water extracts were performed to separate the aqueous (methanol/water) and organic (chloroform) phases. The aqueous phase was lyophilized in a SpeedVac Plus system and was suspended in deuterated water (D_2_O) with 0.04% (v/v) sodium azide, and 0.22 mM 3-(trimethylsilyl)propionic)-2,2,3,3-*d_4_* acid (TSP) was used as chemical shift reference and concentration standard. For extracellular metabolites analysis, 30 μl of 2.2 mM TSP and 30 μl of 0.4% (v/v) sodium azide in D_2_O were added to 540 μl of supernatants. ^1^H NMR spectra were obtained at 25°C in an UltrashieldTM Avance 500 Plus spectrometer (Bruker) equipped with a TCI-Z probe. Spectra were acquired and processed using *TopSpin* 4.1 software (Bruker) and the assignments were made by resorting to spectral databases: Human Metabolome (HMDB) and *Chenomx NMR Suite* 8.11 were used to determine the concentration of the metabolites.

### Immunofluorescence

Immunofluorescence was used to detect the expression of certain proteins. Cells (1 × 10^5^ cells/well) were seeded on glass slides with a 0.2% gelatin coating, until 80% of confluence and then fixed in 2% paraformaldehyde for 15 min at 4°C. After fixation, cells were incubated with 50 mM ammonium chloride (NH_4_Cl) for 10 min. Blocking and antibodies dilutions were performed with PBS 1× 0.5% BSA 0.1% saponin-PBS (w/v/v), between incubations, cells were rinsed twice with PBS 1×, for 5 min. After blocking for 1 h, slides were incubated with rabbit anti-human MCT1 (1:500, ab315382, Abcam) overnight at 4°C. Cells were incubated with Alexa Fluor 488-conjugated goat anti-rabbit (1:1000; A-11008, Invitrogen - Thermo Fisher Scientific) for 2 h at room temperature. The slides were mounted in VECTASHIELD media with DAPI (4′-6-diamidino-2-phenylindole) (Vector Labs) and examined by standard fluorescence microscopy under a Zeiss Imager Z1 AX10 microscope. Images were acquired and processed with *CytoVision* software and quantified with *ImageJ* software.

### Synthesis of PURE_G4_-LA_24_ nanoparticles

Lactic acid-targeted polyurea dendrimer generation four (PURE_G4_-LA_24_) was prepared by reacting polyurea dendrimer generation four (PURE_G4_), obtained using our supercritical-assisted polymerization protocol [33], with activated lactic acid succinic ester (LA-NHS). LA-NHS was synthesized following the literature [34]. Typically, in a round-bottom flask, 41 mg (0.456 mmol) of lactic acid (LA) were dissolved in N,N-Dimethylformamide (DMF) anhydrous (5 ml). After the addition of 105 mg (0.908 mmol) of *N*-hydroxysuccinimide (NHS), 103.6 mg (0.502 mmol) of *N,N*′-dicyclocarbodiimide (DCC), and 0.140 ml (1.04 mmol) of triethylamine (TEA), the reaction was stirred at RT overnight in the dark. After this period, TEA was evaporated and 150 mg of PURE_G4_ (0.019 mmol) in DMF anhydrous (0.75 ml) and 0.070 ml of TEA (0.502 mmol) were added and allowed to react overnight. The mixture was then filtered to remove precipitated solids and TEA was evaporated. The solution was then dialyzed (MWCO 100-500 Da), and the recovered solution was washed several times with diethyl ether to remove residual DMF. After water evaporation, 194 mg (quantitative yield) of the product was obtained as a light-yellow sticky oil. By ^1^H NMR analysis, a total of 24 lactate molecules (50%, PURE_G4_ has 48 available reacting amines at the surface) were incorporated in the dendrimer surface (Supplementary Figure S1).

### Preparation of the SeChry@PURE_G4_-LA_24_ nanoformulation

SeChry was encapsulated in PURE_G4_-LA_24_ nanoparticles following a modified protocol [32]. Briefly, a CHCl_3_ solution (0.5 ml) of SeChry (6.5 mg) was added to an aqueous solution (2 ml) of PURE_G4_-LA_24_ (125 mg). Next, CHCl_3_ was removed in a rotary evaporator and the mixture allowed stirring at RT overnight. Then, the aqueous solution was extracted with CHCl_3_ to remove non-encapsulated SeChry. No SeChry was found in the CHCl_3_ extracts (control by thin-layer chromatography [TLC]), thus confirming a full encapsulation. The release profile followed the usual profile reported for this nanodelivery system.

### Bioinformatic analysis

Lung adenocarcinoma (LUAD) and lung squamous cell carcinoma (LUSC) RNA-Seq data from the Cancer Genome Atlas (TCGA) were analyzed using the cBio Cancer Genomics Portal (http://cbioportal.org, accessed on January 2024) to explore the expression of *SCL16A1* and *SLC16A3* in LUAD and LUSC genomic datasets.

### Statistical analysis

Statistical analysis was performed using *GraphPad Prism* 9.0 software (https://www.graphpad.com). Sample data were presented as the mean (normal distribution) ± SD. Assays were performed with at least three biological replicates per treatment. Comparisons between data from each group were statistically analyzed by a two-tailed unpaired Student’s *t*-test and multiple comparisons were performed using One-way ANOVA with Tukey’s test. For comparisons of two groups, a two-tailed independent-samples *t*-test was used. For the univariate NMR analysis, a Two-way ANOVA with Tukey’s test was used. Differences between experimental conditions were considered statistically significant at *P<*0.05. Multivariate statistical analysis of ^1^H NMR data were performed on *MetaboAnalyst* 5.0 (assessed in November and December 2023) using metabolite concentrations as inputs and scaled using pareto-scaling. Heatmaps representing the univariate analysis of the intracellular and extracellular levels of the different metabolites detected by NMR were created for each cell line using *GraphPad Prism* 9.0 software. For the bioinformatic analysis, a two-tailed unpaired Student’s *t*-test with Welch’s correction was used.

## Results

### SeChry has a profound impact on NSCLC metabolism

**Figure 1:**
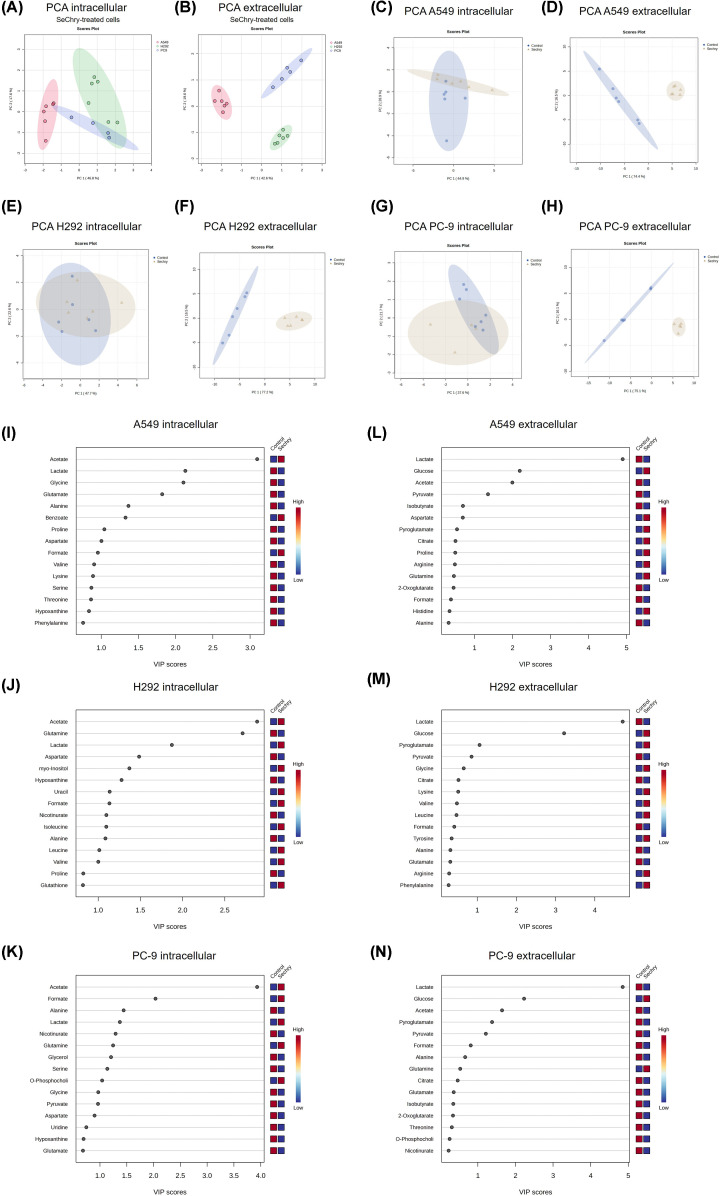
SeChry has a profound impact on key metabolic pathways in NSCLC cells A549, H292 and PC-9 cells were exposed to SeChry for 24 h. Cells were collected, cell extracts were performed, and cell culture media (supernatants) were collected for nuclear magnetic resonance (NMR) spectroscopy analysis. A549, H292 and PC-9 cells were exposed to SeChry for 24 h. Cells were collected, cell extracts were performed, and cell culture media (supernatants) were collected for nuclear magnetic resonance (NMR) spectroscopy analysis. Principal component analysis (PCA) to infer the influence of SeChry on clustering patterns in (**A**) cell extracts and (**B**) supernatant samples of SeChry-treated A549, H292 and PC-9, from ^1^H NMR metabolomic profiles. PCA to infer the influence of SeChry on clustering patterns in (**C,E,G**) cell extracts and (**D,F,H**) supernatant samples of A549, H292 and PC-9, respectively from ^1^H NMR metabolomic profiles. Variable of Importance (VIP) scores plots depicting the 15 most significant metabolites (VIP > 1.0) contributing to the group separations observed in the PLS-DA model in intracellular (**I**) A549, (**J**) H292 (**K**) PC-9 metabolites and in extracellular (**L**) A549, (**M**) H292 and (**N**) PC-9 cells.

The impact of SeChry on cellular metabolism was characterized for A549, H292 and PC-9 cell lines by NMR spectroscopy using the metabolites identified in both supernatants (extracellular) and aqueous fraction of cell extracts (intracellular). In all cell lines, 38 metabolites were identified and quantified, and the specific metabolic patterns were assessed using principal component analysis (PCA) and partial least-squares discriminant analysis (PLS-DA). A PCA was conducted using samples from all cell lines exposed to SeChry. In PCA analysis, the first principal component (PC1) captures the greatest amount of variance in the data, while the second principal component (PC2) captures the second greatest amount of variance. In intracellular extracts, an overlap between H292 and PC-9 cells suggested that they share similar endometabolomes (Figure 1A). PC1 explains 46.8% of the total variance, while PC2 explains 17.8%. Conversely, extracellular extracts exhibited a distinct separation among the three cell lines, where PC1 explains 42.6% of the total variance and PC2 explains 19.3% (Figure 1B). PLS-DA was also performed, confirming clear separations in both intracellular and extracellular samples exposed to SeChry (Supplementary Figure S2C and E). The predictive ability of the PLS-DA model to discriminate between the three cell lines exposed to SeChry was evaluated by adopting the criteria for success of R^2^ ≥ 0.7 and Q^2^ ≥ 0.4 [33]. For intracellular samples, the generated models achieved an *R*^2^ of 0.99 and Q^2^ of 0.91 and for extracellular samples, the *R*^2^ was 0.96, and *Q*^2^ was 0.91, demonstrating robust model performance in both intra and extracellular contexts (Supplementary Figure S2D and F).

Moreover, a PCA to compare the control samples and SeChry samples in each cell line was also conducted. In intracellular extracts from all cell lines, there was some overlap between the control and SeChry-treated cells, especially in H292 cells; however, a clear separation was evident in supernatant samples (Figure 1C–H). Regarding the PCA plots of intracellular metabolites, A549 cells show PC1 explaining 44.9% and PC2 explaining 23.0% of the variance (Figure 1C), H292 cells show PC1 explaining 47.7% and PC2 explaining 20.0% of the variance (Figure 1E), and PC-9 cells show PC1 explaining 37.6% and PC2 explaining 21.9% of the variance (Figure 1G). For extracellular metabolites, the PCA plots reveal PC1 explaining 74.4% and PC2 explaining 12.1% of the variance in A549 cells (Figure 1D), PC1 explaining 77.4% and PC2 explaining 13.5% of the variance in H292 cells (Figure 1F), and PC1 explaining 75.1% and PC2 explaining 16.1% of the variance in PC-9 cells (Figure 1H).

The PLS-DA analysis revealed better discrimination between the two groups in all cell lines, being most evident in PC-9 cells (Supplementary Figure S2G, I, K, M, O and Q). Regarding intracellular samples, the present models obtained an *R*^2^ of 0.88 and *Q*^2^ of 0.62 for A549 samples, an *R*^2^ of 0.99 and *Q*^2^ of 0.86 for H292 samples and an *R*^2^ of 0.99 and *Q*^2^ of 0.84 for PC-9 samples, demonstrating its suitability for discriminating between control and SeChry-treated samples (Supplementary Figure S2H, L and P). As for extracellular samples, the models obtained an *R*^2^ of 0.99 and *Q*^2^ of 0.98 for A549 samples, an *R*^2^ of 0.99 and *Q*^2^ of 0.98 for H292 samples and an *R*^2^ of 0.99 and *Q*^2^ of 0.99 for PC-9 samples, clearly showing its capacity to differentiate between control and SeChry-treated samples (Supplementary Figure S2J, N and R). Permutation tests from PLS-DA models did not show significant *p*-values, probably due to the small sample size assessed (data not shown).

The variable importance in projection (VIP) scores of PLS-DA were calculated to discern the metabolites contributing significantly to the segregation between control and SeChry-treated groups. A VIP score greater than 1 is the typical rule for selecting relevant variables [34]. In A549 cells, acetate, lactate, glycine, glutamate, and alanine emerged as pivotal metabolites for the observed separation (Figure 1I). H292 cells exhibited a distinct metabolic fingerprint, with acetate, glutamine, lactate, aspartate, and myo-inositol playing key roles in the discrimination (Figure 1J). Similarly, in PC-9 cells, the metabolites acetate, formate, alanine, lactate, and nicotinurate were identified as significant contributors to the observed separation (Figure 1K). In extracellular samples, the metabolites driving the separation in A549 cells included lactate, glucose, acetate, and pyruvate (Figure 1L). For H292 cells, the differentiating metabolites were lactate, glucose, and pyroglutamate, while in PC-9 cells, the contributing metabolites were lactate, glucose, acetate, pyroglutamate, and pyruvate (Figure 1M,N).

### SeChry treatment induces distinct yet overlapping alterations in key metabolic pathways across NSCLC cell lines

**Figure 2:**
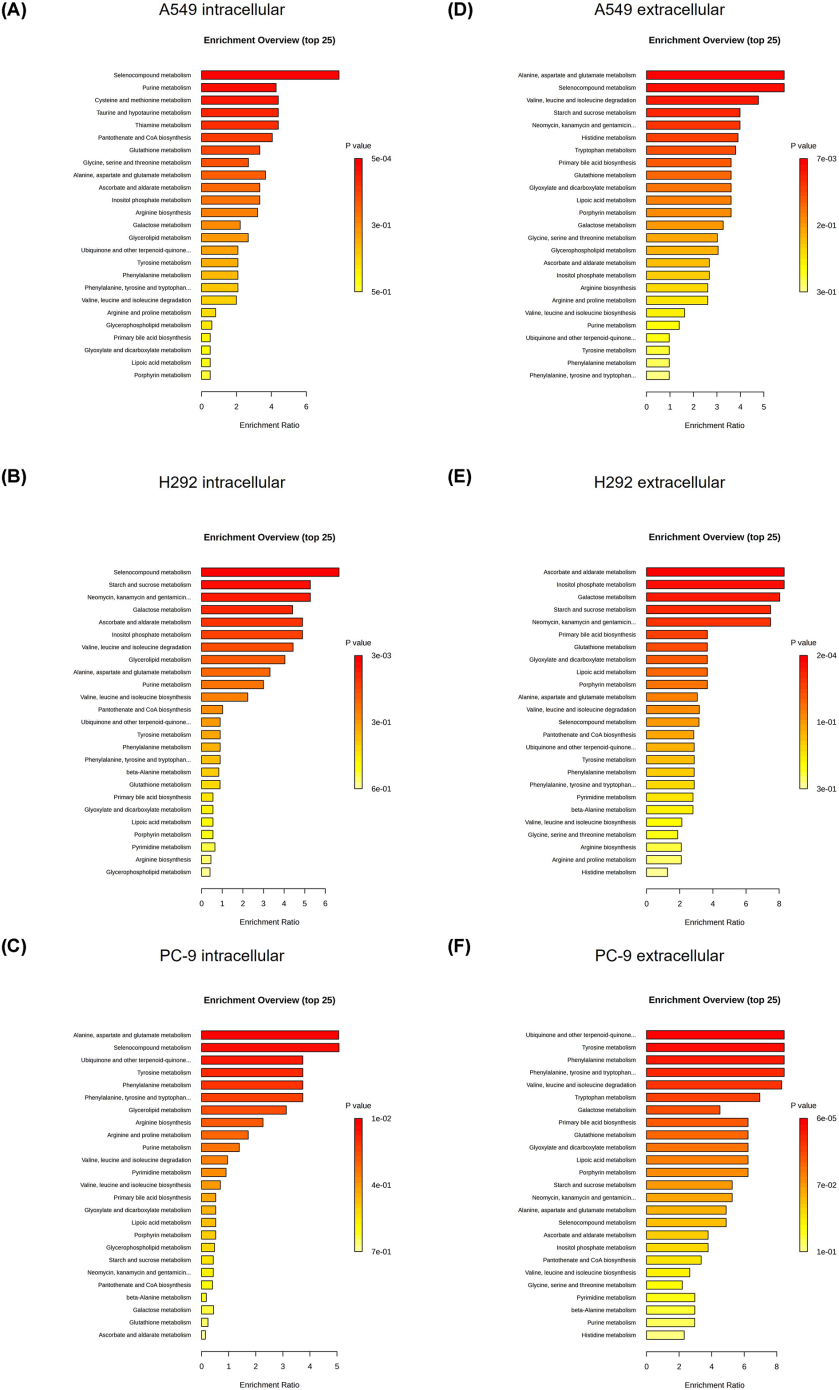
SeChry induces metabolic shifts predominantly involving selenocompound metabolism Metabolite set enrichment analysis (MSEA) to pinpoint the pathways influenced by SeChry in intracellular (**A**) A549, (**B**) H292 and (**C**) PC-9 metabolites and in extracellular (**D**) A549, (**E**) H292 and (**F**) PC-9 metabolites. The enrichment ratio is calculated as the number of hits within a particular metabolic pathway divided by the expected number of hits.

To further clarify alterations in metabolism and to pinpoint the pathways influenced by SeChry, we conducted metabolite set enrichment analysis (MSEA). The SeChry-induced metabolic shifts predominantly involved selenocompound metabolism at the intracellular level across all cell lines (Figure 2A–C). In PC-9 cells, alanine, aspartate and glutamate metabolism was also altered. At the extracellular level, A549 cells exhibited notable changes in alanine, aspartate, and glutamate metabolism; selenocompound metabolism; and valine, leucine, and isoleucine degradation (Figure 2D). H292 featured enrichments in ascorbate and aldarate metabolism; inositol phosphate metabolism and galactose metabolism (Figure 2E). PC-9 demonstrated enhancements in ubiquinone and other terpenoid-quinone biosynthesis; tyrosine metabolism; phenylalanine metabolism; phenylalanine, tyrosine, and tryptophan biosynthesis; and valine, leucine, and isoleucine degradation (Figure 2F).

We also conducted metabolic pathway enrichment analysis for control versus SeChry-treated cells. The pathway library (KEGG database) comprised 80 known human metabolic pathways [35]. This analysis showed that some similarities in the most impacted metabolic pathways were found in the three assessed cell lines. Still, differences in each cell type were also revealed and are detailed below (Supplementary Figure S3A–F). In both intra- and extracellular samples, glycolysis/gluconeogenesis was significantly impacted by the SeChry treatment in all cell lines. Moreover, arginine biosynthesis; cysteine and methionine metabolism; alanine, aspartate and glutamate metabolism and the TCA cycle were significantly influenced by SeChry in the extracellular media of A549, H292 and PC-9 cells.

In A549 intracellular samples, SeChry significantly impacted nine metabolic pathways, with the most affected being purine metabolism, cysteine and methionine metabolism, and pyruvate metabolism (Supplementary Figure S3A). Extracellular samples from A549 cells revealed significant alterations in 12 pathways, with the top three being butanoate metabolism, arginine biosynthesis, and alanine, aspartate, and glutamate metabolism (Supplementary Figure S3B). In H292 cells, intracellular samples showed changes in two pathways-inositol phosphate metabolism and glycerolipid metabolism (Supplementary Figure S3C). Extracellular samples from H292 cells displayed significant disturbances in 14 metabolic pathways, with the most affected being alanine, aspartate, and glutamate metabolism, TCA cycle, and arginine biosynthesis (Supplementary Figure S3D). For PC-9 cells, SeChry significantly disturbed 11 pathways in intracellular samples, including tyrosine metabolism, TCA cycle, and cysteine and methionine metabolism (Supplementary Figure S3E). Supernatants from PC-9 cells exhibited alterations in 17 metabolic pathways, with the top three being alanine, aspartate, and glutamate metabolism, cysteine and methionine metabolism, and phenylalanine, tyrosine, and tryptophan biosynthesis (Supplementary Figure S3F).

### SeChry induces similar patterns of elevation and reduction in metabolite levels across NSCLC cell lines

**Figure 3:**
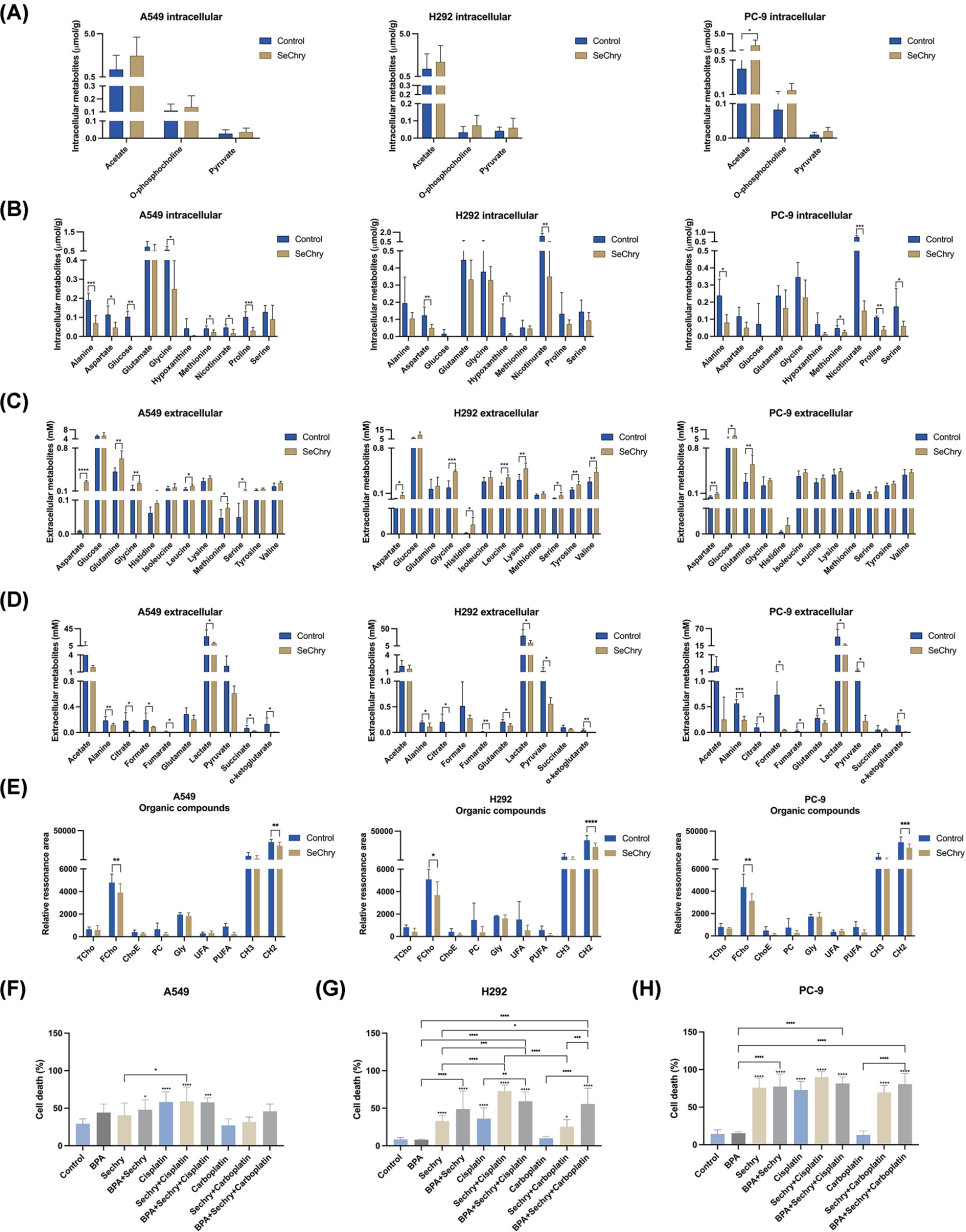
SeChry triggers NSCLC cell lines’ metabolic profiles and serves as an effective cancer therapy A549, H292 and PC-9 cells were exposed to SeChry for 24 h. Cells were collected, cell extracts were performed, and cell culture media (supernatants) were collected for nuclear magnetic resonance (NMR) spectroscopy analysis. Levels of (**A**) increased and (**B**) decreased intracellular metabolites in A549, H292, PC-9 cells. Levels of (**C**) increased and (**D**) decreased extracellular metabolites in A549, H292, PC-9 cells. (**E**) Identified functional groups and lipid constituents in organic samples. TCho, Total cholesterol; FCho, Free cholesterol, ChoE- Esterified cholesterol; PC, Phosphatidylcholine; Gly, Glycerol; UFA, Unsaturated fatty acid; (-CH = CH-); PUFA, Polyunsaturated fatty acid (-CH = CH-CH2-CH = CH-), CH3, methyl group of fatty acids; CH2, methylene group of fatty acids. Data is represented as mean ± SD. *P<0.5, **P<0.01, ***P<0.001, ****P<0.0001 (two-tailed unpaired Student’s *t*-test). To evaluate the cooperation between SeChry and other cytotoxic compounds, A549, H292 and PC-9 cells were exposed to SeChry for 48 h, and in the last 24 h, BPA, cisplatin or carboplatin were added. Percentage of cell death analyzed by flow cytometry using Annexin V and PI in (**F**) A549, (**G**) H292 and (**H**) PC-9 cells. Data are represented as mean ± SD. *P<0.5, **P<0.01, ***P<0.001, ****P<0.0001 (one-way ANOVA with Tukey’s multiple comparisons test was used).

To better understand which specific metabolites were altered by SeChry, univariate analysis of the intracellular and extracellular levels of the different metabolites detected by NMR was performed using an independent samples *t*-test to compare the differences between control and SeChry-treated cells. SeChry led to similar trends in increasing or decreasing some metabolites in the three cell lines (Figure 3). At the intracellular level, SeChry demonstrated a tendency to increase acetate, *O*-phosphocholine, and pyruvate levels in all cell lines; however, acetate was significantly increased specifically in PC-9 cells (Figure 3A). The metabolites that tended to be decreased by SeChry included the amino acids alanine, aspartate, glutamate, glycine, methionine, proline, and serine (Figure 3B). Moreover, glucose and hypoxanthine also tended to be decreased by SeChry. Alanine, methionine and proline were significantly decreased by SeChry in A549 and PC-9. Glycine and serine levels are significantly lower in A549 and PC-9 cells, respectively. In H292 intracellular extracts, SeChry significantly reduced the levels of aspartate and hypoxanthine. Nicotinurate levels were diminished in all cell lines in SeChry presence.

SeChry led to a significant increase of aspartate and glucose extracellular levels in all cell lines (Figure 3C), suggesting that there was decreased consumption of aspartate and glucose. There was also a trend to decrease the consumption of the amino acids glutamine, glycine, histidine, isoleucine, leucine, lysine, methionine, serine, tyrosine, and valine (Figure 3C). Additionally, A549 and H292 cells exhibited elevated extracellular levels of glycine, leucine, and serine after SeChry exposure, while A549 and PC-9 cells showed enhanced glutamine. Moreover, A549 SeChry-treated samples displayed significantly higher extracellular methionine levels, and H292 cells experienced increases in histidine, tyrosine, and valine. SeChry induced a trend toward decreased levels of extracellular metabolites, including organic acids such as acetate, citrate, formate, fumarate, lactate, pyruvate, succinate, and α-ketoglutarate (Figure 3D). Similarly, amino acids such as alanine and glutamate also exhibited a reduction. Alanine, citrate, fumarate, lactate and α-ketoglutarate levels were significantly lower in all cell lines. Moreover, formate levels decreased in A549 and PC-9 cells, while pyruvate levels were reduced in H292 and PC-9 cells. Thus, SeChry led to the depletion of many amino acids, which are needed to reduce oxidative stress and support cell survival. The organic phases of the cell extracts were also analyzed, and decreased levels of free cholesterol (FCho) and methylene groups of fatty acids (CH2) were found after SeChry exposure in all cell lines (Figure 3E). A tendency to decrease phosphatidylcholine (PC) and polyunsaturated fatty acids (PUFAs) was also seen, concomitantly with a decrease in lipid unsaturation (Supplementary Figures S4G, 5G and 6G).

A two-way ANOVA with Tukey’s test was also employed to compare the differences between control and SeChry-treated cells in intracellular amino acids and peptides, intracellular sugars and organic acids and other intracellular compounds groups. In the A549 cell line, SeChry treatment led to a significant decrease in intracellular levels of glutamate and glycine (Supplementary Figure S4A). The extracellular media (supernatants) showed a notable increase in arginine, aspartate, glutamine, and proline post-SeChry exposure (Supplementary Figure S4D). Moreover, extracellular lactate levels were reduced (Supplementary Figure 4E). In H292 cells, SeChry treatment resulted in a significant drop in intracellular levels of glutamine and nicotinurate (Supplementary Figure S5A and C). In contrast, extracellular glycine, leucine, lysine, and valine experienced a significant increase after SeChry exposure (Supplementary Figure S5D). Similar to A549, H292 cells exhibited reduced extracellular lactate and nicotinurate levels (Supplementary Figure S5E and F). For PC-9 cells, the SeChry impact was evident with a decrease in intracellular alanine, glycine, and nicotinurate (Supplementary Figure S6A and C). Intriguingly, there was a rise in intracellular acetate and *O*-phosphocholine (Supplementary Figure S6B and C). Extracellular metabolites told a compelling tale as well, with lower levels of alanine and lactate, *o*-phosphocholine, and uracil following SeChry exposure. Interestingly, threonine and pyroglutamate decreased, but glutamine levels increased (Supplementary Figure S6D–F).

### SeChry may serve as a beneficial adjunct in cancer therapy

Cancer cells may utilize lactate as a fuel source by importing it through MCT1. Subsequently, lactate is converted into pyruvate by LDHB to sustain oxidative phosphorylation (OXPHOS) [24]. We recently, published that SeChry induces NSCLC cell death [21], and the consumption of lactate is pivotal in NSCLC cells [22]. Thus, next, we aimed to disclose the impact of SeChry on cell death upon BPA, and chemotherapeutic agents’ exposure in NSCLC. The analog of lactate, BPA is a potent cytotoxic drug that inhibits glycolysis, reducing cellular energy levels, having been proposed as a therapeutic alternative as cancer cells can import it through MCT1 [36,37]. For that, cells were treated with SeChry for 48 h, and in the last 24 h, BPA, cisplatin, or carboplatin were added. Figure 3F–H shows that all cell lines are sensitive to cisplatin and resistant to carboplatin (when administered alone).

In A549 cells, cisplatin significantly increased cell death, as well as SeChry in combination with BPA with or without cisplatin (Figure 3F). The combinatory treatment with carboplatin did not have any effect. In H292 and PC-9 cells, SeChry showed higher toxicity and its combination with BPA, cisplatin or carboplatin further enhanced it (Figure 3G,H). These differences were most prominent in H292 cells, and the most interesting significant effect was observed when cells were exposed to BPA, SeChry and carboplatin (Figure 3G). The combination of SeChry with BPA appears to have a more noticeable effect on H292 cell line. PC-9 cells showed the highest sensitivity to SeChry, though the combined effect was less pronounced compared with H292. Nonetheless, these findings suggest that SeChry could act as a beneficial adjunct in cancer therapy.

### SeChry@PURE-_G4_-LA_24_ exhibits selective cytotoxicity against NSCLC cell lines, with varying sensitivity across different molecular backgrounds

**Figure 4:**
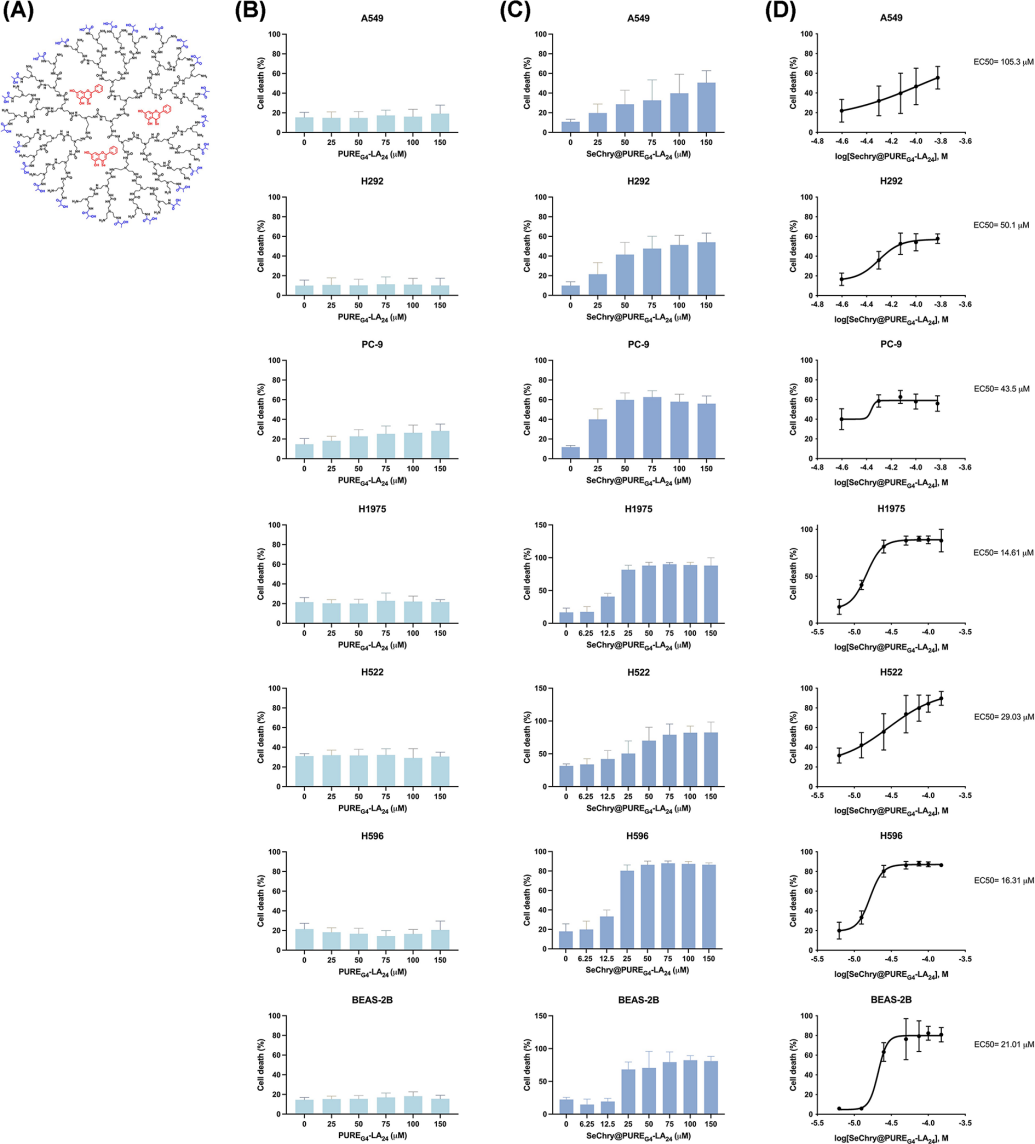
Sechry@PUREG4-LA24 shows specific toxicity against NSCLC cell lines (**A**) SeChry@PURE_G4_-LA_24_ nanoformulation. Chemical structures of the generation four polyurea dendrimer lactate conjugate and encapsulated SeChry molecules. A549, H292, PC-9, H1975, H522, H596 and BEAS-2B cells were exposed to empty nanoparticles (PURE_G4_-LA) or SeChry@PURE_G4_-LA_24_ for 48 h. Percentage of cell death analyzed by flow cytometry using Annexin V and PI in (**B**) PURE_G4_-LA_24_-treated cells and (**C**) Sechry@PURE_G4_-LA_24_-treated cells. (**D**) EC50 curves and values for SeChry@PURE_G4_-LA. Data are represented as mean ± SD. *P<0.5, **P<0.01, ***P<0.001, ****P<0.0001 (one-way ANOVA with Tukey’s multiple comparisons test was used).

We previously showed that SeChry increased oxidative stress prompting GSH depletion, and inhibition of the H_2_S-generating enzyme CBS [17]. However, this compound was too toxic for non-malignant cells, thus the nanoparticles were functionalized with folate to increase the specificity to cancer cells, which have high expression levels of the folate receptor [17]. Very promising results were obtained in ovarian cancer [17], consequently, we assessed the efficacy of this nanoformulation, but now using dendrimer nanoparticles functionalized with lactic acid, SeChry@PURE_G4_-LA_24_ (Figure 4A), given our observation of NSCLC’s dependency on lactate [22].

The cell lines were exposed to different concentrations (6.25–150 µM) of SeChry@PURE_G4_-LA_24_ (nanoencapsulated SeChry) and empty PURE_G4_-LA_24_ for 48 h and the EC_50_ was determined. Here, to confirm the reliability of these formulations across different NSCLC cell lines with different molecular backgrounds, three additional cell lines were examined (H1975, H522, H596) and BEAS-2B cells were used as a non-malignant control. The empty nanoformulation did not induce toxicity in any of the cell lines (Figure 4B). Regarding the cytotoxic response to SeChry@PURE_G4_-LA_24_, the least sensitive cells were the A549 cells with an EC_50_ = 105.3 µM (Figure 4C,D). These were followed by the H292 cells (EC_50_ = 50.1 µM), PC-9 cells (EC_50_ = 43.5 µM), H522 (EC_50_ = 29.03 µM), BEAS-2B (EC = 21.01 µM), H596 (EC_50_ = 16.31 µM) and H1975 (EC_50_ = 14.61 µM) (Figure 4C,D).

A549 and H292 cells treated with SeChry predominantly underwent a combination of early and late apoptosis. PC-9 cells treated with SeChry primarily exhibited necrosis, along with early and late apoptosis, with only a small percentage showing early apoptosis (data not shown). In contrast, treatment with SeChry@PURE_G4_-LA_24_ resulted predominantly in necrosis and necroptosis across all studied cell lines (data not shown). Further detailed analysis of the types of cell death will be addressed in the future.

**Figure 5:**
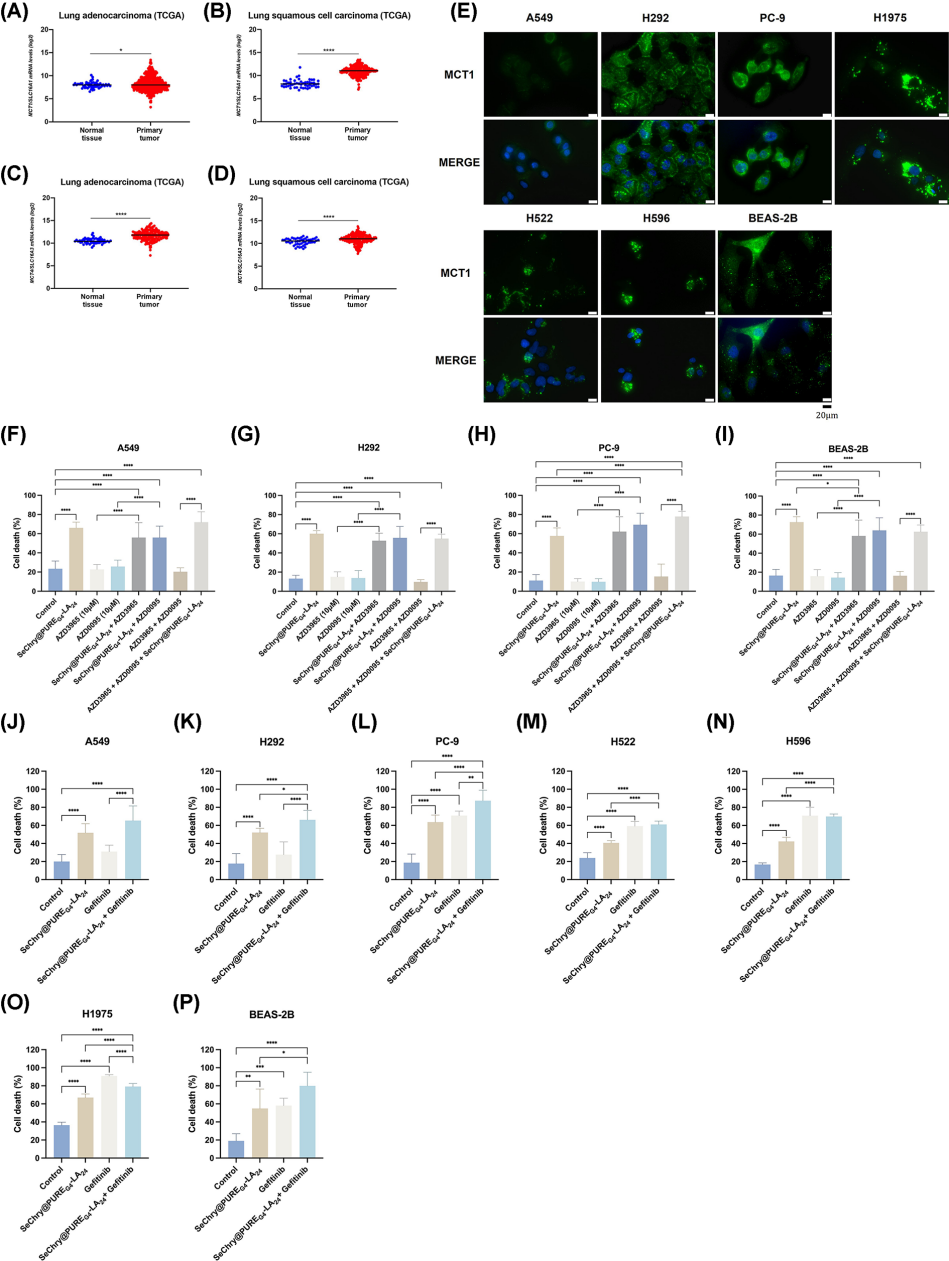
*MCT1/SLC16A1* and *MCT4/SLC16A3* expression is increased in NSCLC, and targeting MCTs may offer a viable alternative for combination therapy Analysis of the expression profiles of *MCT1/SLC16A1* in normal lung tissue and primary carcinomas in (**A**) LUAD and (**B**) LUSC and *MCT4/SLC16A3* in (**C**) LUAD and (**D**) LUSC extracted from the TCGA database. A two-tailed unpaired Student’s *t*-test with Welch’s correction was used. (**E**) Cells were kept in control conditions and immunofluorescence analysis showed that all cell lines express MCT1. Nuclei were stained with DAPI (blue). The white bars scale means 20 μm. The cytotoxic response to SeChry@PURE_G4_-LA_24_ combined with MCT1 or MCT4 inhibitors was evaluated. Cells were incubated overnight with the MCT1 inhibitor AZD3965 and/or the MCT4 inhibitor AZD0095 and the following day, they were treated with SeChry@PURE_G4_-LA_24_ at the determined LC50 concentration. Percentage of cell death analyzed by flow cytometry using Annexin V and PI in (**F**) A549, (**G**) H292, (**H**) PC-9 and (**I**) BEAS-2B cells. Data are represented as mean ± SD. *P<0.5, **P<0.01, ***P<0.001, ****P<0.0001 (one-way ANOVA with Tukey’s multiple comparisons test was used). The effect of the combination of SeChry@PURE_G4_-LA_24_ with the EGFR inhibitor gefitinib was evaluated. Cells, (**J**) A549, (**K**) H292, (**L**) PC-9, (**M**) H522, (**N**) H596, (**O**) H1975, and (**P**) BEAS, were incubated overnight with gefitinib and the following day, they were treated with SeChry@PURE_G4_-LA_24_ at the determined LC50 concentration. Data are represented as mean ± SD. *P<0.5, **P<0.01, ***P<0.001, ****P<0.0001 (one-way ANOVA with Tukey’s multiple comparisons test was used).

Although lactate was initially characterized as a waste product of anaerobic metabolism, it is now widely recognized that under fully aerobic conditions, various cells continuously utilize lactate as a fuel source [38]. Indeed, we showed that NSCLC cells exhibit increased lactate production and consumption [22]. MCT1 and MCT4 are essential players in the process of lactate exchange within tumors. Despite being able to transport lactate in both directions across the cell membrane, MCT1 is more associated with lactate import while MCT4 is linked to lactate export [24]. MCT1 and MCT4 overexpression has been identified in various cancer types, including NSCLC, and is associated with poor outcomes [39,40]. Moreover, the expression of MCT1 and MCT4 is negative/low in adjacent non-tumor lung tissues [39]. We examined TCGA transcriptomic data for the two predominant NSCLC groups, LUAD and LUSC, along with normal lung tissue, to assess the expression patterns of *SLC16A1* (encoding MCT1) and *SLC16A3* (encoding MCT4). Both *SLC16A1* and *SLC16A3* exhibit significant up-regulation in both LUAD (Figure 5A,C) and LUSC (Figure 5B,D) compared with normal tissue. MCT1 levels were assessed by immunofluorescence to confirm the capacity to take up lactate by the previous cell lines. The least sensitive cells (A549 cells) were the ones showing the lowest levels of MCT1 (Figure 5E). H1975, H292, PC-9, H522, H596, and BEAS-2B cells show high MCT1 expression (Figure 5E) consistent with the increased sensitivity to SeChry@PURE_G4_-LA_24_ (Figure 4).

### The cytotoxic response to SeChry@PURE_G4_-LA_24_ with MCT1 or MCT4 inhibitors varies depending on the specific cell line

Subsequently, we aimed to determine whether inhibiting MCT1 or MCT4 would reduce the sensitivity to SeChry@PURE_G4_-LA_24_. Cells were incubated overnight with the MCT1 inhibitor AZD3965 and/or the MCT4 inhibitor AZD0095. After, they were treated with SeChry@PURE_G4_-LA_24_ at the determined EC_50_ concentration, for 24 h. The inhibitors, when administered alone or in combination, did not induce any toxicity in any of the cell lines (Figure 5F–I). SeChry@PURE_G4_-LA_24_ in combination with either AZD3965 or AZD0095 (alone or combined) significantly increased cell death when compared with the control (Figure 5F-I). The double inhibition with AZD3965 plus AZD0095 combined with SeChry@PURE_G4_-LA_24_ further enhanced cell death in PC-9 cells (Figure 5H). When comparing to SeChry@PURE_G4_-LA_24_ treatment, a small tendency to decrease cell death was observed when SeChry@PURE_G4_-LA_24_ was combined with AZD3965 and AZD0095 in A549 cells and H292 cells (Figure 5F,G). While in PC-9, MCT1 or MCT4 inhibition in conjunction with SeChry@PURE_G4_-LA_24_ tended to increase cell death levels (Figure 5H). In BEAS-2B cells, SeChry@PURE_G4_-LA_24_ plus MCT1 inhibitor AZD3965 significantly decreased cell death in comparison with SeChry@PURE_G4_-LA_24_ treatment (Figure 5I).

Given the fundamental role of EGFR signaling in NSCLC [22], the combination of SeChry@PURE_G4_-LA_24_ plus gefitinib was also explored (Figure 5J–P). The most sensitive cell lines to gefitinib were the H1975, followed by H596, PC-9, H522 and BEAS-2B (Figure 5L-N,P). In all cell lines except for A549, SeChry@PURE_G4_-LA_24_ combined with gefitinib significantly increased cell death when compared with SeChry@PURE_G4_-LA_24_ treatment alone (Figure 5K-P).

Comparing with gefitinib alone, the combined treatment with SeChry@PURE_G4_-LA_24_ further increased cell death in A549, H292, PC-9 and BEAS-2B (Figure 5J-L,P), while in H1975, the opposite was observed (Figure 5O). Moreover, no meaningful differences were found in H522 and H596 (Figure 5M,N). Therefore, in some NSCLC subsets the combination of SeChry@PURE_G4_-LA_24_ with targeted therapy can be an alternative to improve therapy.

## Discussion

In the present study, we aimed to thoroughly characterize the metabolism of SeChry and elucidate the mechanisms responsible for its cytotoxic effects in NSCLC. SeChry treatment had a notable impact on the metabolism of NSCLC cell lines and caused distinct yet overlapping changes in crucial metabolic pathways (Figures 1 and 2; Supplementary Figure S3). We found that SeChry induced metabolic shifts predominantly involved selenocompound metabolism at the intracellular level across all cell lines (Figure 2A-C). Selenium is an essential trace element that has attracted interest due to its antioxidant and anti-cancer properties [41]. Selenium functioning as an antioxidant, exhibits significant potential for regulating redox processes and maintaining cellular homeostasis and metabolism. It is metabolized and incorporated as the amino acid selenocysteine (Sec) and is required for the proper enzymatic function of selenoproteins such as glutathione peroxidases (GPX) and thioredoxin reductases (TrxR) [42,43]. Conversely, at higher doses, selenium functions as a pro-oxidant by oxidizing thiols and generating ROS, leading to cytotoxicity in cells [44,45].

Inadequate selenium levels in cells may activate a selenium recycling mechanism, orchestrated by the enzyme selenocysteine-lyase (SCLY) [42,46]. This enzyme decomposes selenocysteine into hydrogen selenide (H_2_Se) and alanine [46], which can then be recycled for reuse in selenoprotein synthesis. According to the Metaboanalyst analysis, SeChry impacts selenocompound metabolism through the decrease in alanine levels, which occurred in the three cell lines (Figure 3B). Cancer cells rely on amino acids for survival, particularly in the presence of genotoxic, oxidative, and nutritional stress conditions [47]. Indeed, alanine can serve as an energy source during fasting conditions through the conversion to pyruvate for glucose synthesis via the gluconeogenesis pathway [48], and conversion of alanine to pyruvate, entering the TCA cycle [48]. Glycolysis/gluconeogenesis and the TCA cycle were both impacted by SeChry in all the cell lines (Supplementary Figure S3A–F). As alanine levels diminished in both intra and extracellular samples, the above-mentioned pathways are likely being hindered by SeChry.

Acetate levels tended to be higher in the cell extracts and lower in the supernatants in the presence of SeChry, indicating a decrease in the release of this metabolite by NSCLC cells (Figure 3A,D). Besides this, acetate was the most important metabolite contributing to the separation between the control and SeChry-treated cells (Figure 2I–K). Acetate is conjugated with coenzyme A (CoA) through acetyl-CoA synthetases (ACSSs) to generate acetyl-CoA, a compound utilized in both fatty acid synthesis (lipogenesis), which is required for cell growth and survival, and histone acetylation [49]. Cancer cells utilize acetate as a nutritional source in an ACSS2-dependent manner, supporting the biosynthesis of membrane phospholipids [50]. Accordingly, intracellular *O*-phosphocholine levels also tended to increase after SeChry exposure, while extracellular levels were decreased in H292 and PC-9 cells (Figure 3A). Thus, in this context, it is likely that acetate is not being utilized by the NSCLC cells exposed to SeChry, leading to its intracellular accumulation in the cell. As acetate is not converted into acetyl-CoA, essential biosynthetic processes for nucleotides, amino acids, fatty acids, and cholesterol are hindered, thus leading to impaired cell survival and growth.

Exposure to SeChry resulted in alterations in lipid composition, notably a decrease in FCho, PC, PUFAs, and overall lipid unsaturation (Figure 3E and Supplementary Figures S4, 5 and 6G). Lipid peroxidation, a hallmark of ferroptosis, is a process in which oxidants, such as free radicals, attack lipids containing carbon-carbon double bonds, especially PUFAs [51,52]. Thus, the observed changes in lipid metabolism may have important implications for lipid peroxidation and ferroptosis. The reduction in PUFAs disrupts the cellular lipid milieu, thereby intensifying the cells’ susceptibility to oxidative stress and ferroptosis. Furthermore, decreased lipid unsaturation leads to increased membrane rigidity, affecting fluidity and the functional integrity of transporters and enzymes that maintain redox balance [53], sensitizing cells to ferroptosis.

Another significant metabolite contributing to the major impact of SeChry on NSLSC metabolism was lactate (Figure 1I–N). In all cell lines, extracellular lactate was significantly reduced by SeChry, and extracellular glucose was increased, indicating that glucose catabolism is impaired (Figure 3D). This is supported by the undetectable intracellular levels of glucose upon SeChry exposure (Figure 3B). Moreover, in H292 and PC-9 cells, lactate levels tended to increase, suggesting an increase in the uptake of this metabolite and consequent accumulation (Supplementary Figures S5B and 6B). Despite histological and genetic diversity, human lung tumors utilize both glycolytic and OXPHOS pathways concurrently for glucose oxidation [54]. Lactate cannot accumulate within the cell to avoid a decrease in cytoplasmic pH, which could impact cell viability [55]. Therefore, the up-regulation of MCTs, facilitating the transport of lactate across the cell membrane to the extracellular medium, is a common phenomenon in cancer cells [24,55]. Thus, it appears that H292 and PC-9 cells exposed to SeChry are effectively uptaking lactate, since they express high levels of MCT1 (Figure 5E), but SeChry might hinder their capacity to export lactate, leading to its accumulation and concurrent loss of cell viability. This accumulation disrupts pH homeostasis, ultimately inducing intracellular acidification and triggering cancer cell death. In A549 cells, the intracellular accumulation of lactate is not observed (Supplementary Figure S4A), which may be related to the fact that these cells show lower levels of MCT1. Thus, other metabolic alterations are inducing cell death in A549 cells. This difference in response could be a contributing factor to the observed metabolic similarities between H292 and PC-9 cells, distinct from the response seen in A549 cells (Figure 1A). Indeed, the metabolites that promoted the highest separation between the three cell lines exposed to SeChry were cysteine (intracellularly), which is only present in A549 cells, and histidine (extracellularly), showing the highest levels also in A549 (Supplementary Figure S2A and B). Dynamin-related protein (DRP1) enhances lactate utilization in *KRAS* mutant NSCLC cells by reducing ROS production, protecting cells from oxidative damage [56]. Inhibition of DRP1 disrupts redox control, suppresses lactate utilization, and increases susceptibility to ROS-induced apoptosis. Similarly, SeChry exposure impairs lactate utilization in *KRAS* mutant A549 cells, suggesting that SeChry-related cell death in A549 cells may not involve necroptosis.

Here, we found that SeChry led to a significant increase in glucose extracellular levels, while it tended to reduce it at the intracellular level (Figure 3B,C). This suggests that SeChry may hinder the consumption of glucose. In healthy cells, glucose is transported through glucose transporters (GLUT1-14) and undergoes glycolysis, resulting in the production of pyruvate [57]. Intracellular pyruvate levels were not significantly altered by SeChry (Supplementary Figures S4 and 5). Thus, although glycolysis remains active, it seems that NSCLC cells exhibit a reduced reliance on this pathway.

We previously demonstrated that SeChry promoted GSH depletion, being probably associated with an increase in oxidative stress [17]. Here, we found that GSH levels tended to be lower in A549 and PC-9 cells but higher in H292 cells after SeChry treatment (Supplementary Figures S4A, 5A and 6A). The higher levels of GSH in H292 may be explained by the very high intracellular levels of glutamine/glutamate or might indicate that these cells are slightly more resistant to SeChry than A549 and PC-9 cells, as they show the lowest levels of cell death (Figure 3G). Moreover, the intracellular levels of amino acids involved in GSH production, such as glutamate, glycine and methionine were also reduced (Figure 3B). Cysteine was not detected in H292 or PC-9 cells, but only in A549, suggesting that H292 and PC-9 cells rely more on cysteine compared with A549 (Supplementary Figure S4A). The elevated intracellular glutamate levels in A549 cells also highlight that these cells utilize less glutamate for cysteine import compared with H292 and PC-9 cells. This is attributed to the exchange of glutamate with cystine through the xCT antiporter, expressed in all the examined cell lines. The fact that glutamate decreased both intra- and extracellularly might suggest that SeChry affects the xCT transporter. Moreover, in PC-9 cells, SeChry seemed to induce an increase in the uptake of pyroglutamate (Supplementary Figure S6A and D), an intermediate in GSH metabolism, which was reported to be associated with GSH depletion and oxidative stress when detected in high levels [58,59].

The pyrimidine nucleosides uridine and deoxyuridine have emerged as powerful relievers of oxidative stress [60]. H292 cells showed a trend to increase the synthesis of nitrogenous bases, such as purine synthesis (guanosine) and pyrimidine (cytidine, uracil, uridine) indicating that they might adapt to conditions of oxidative stress, at least the cells that are surviving (Supplementary Figure S5C). In cellular response to oxidative stress, the Nrf2 signaling cascade, regulated by inositol polyphosphate multikinase (IPMK), plays a pivotal role in maintaining redox homeostasis [61,62]. IPMK binding to Nrf2 inhibits its activation and the expression of target genes. Depletion of IPMK enhances GSH and cysteine levels, leading to heightened resistance against oxidants [61]. Interestingly, SeChry-treated H292 cells showed alterations in the inositol phosphate pathway metabolism, as myo-inositol levels increased (Figure 1J).

Amino acids linked to the TCA cycle, such as alanine, glutamate, and aspartate, some of which are involved in ammonia clearance (glutamate and alanine), also tended to be depleted by SeChry (Figure 3B). Moreover, GSH metabolism; cysteine and methionine; and the TCA cycle were all significantly impacted by SeChry (Supplementary Figure S3). SeChry likely induces oxidative stress and cell death by depleting GSH through the loss of essential amino acids. These amino acids are crucial for GSH production and regulate cell proliferation and survival. For instance, glycine promotes the growth of cancer cells by enhancing the antioxidant pool and participating in one-carbon metabolism crucial for nucleotide biosynthesis [63,64]. Similarly, glutamate acts as an anaplerotic substrate for the TCA cycle and functions as a detoxifying agent for free ammonia [65]. The methionine cycle is vital in maintaining tetrahydrofolate (THF4) levels, central to one-carbon metabolism that supports nucleotide synthesis and cancer cell proliferation [66]. Glutamine-derived aspartate has been recognized as crucial for nucleotide biosynthesis and redox control in cancer cells [67]. TCA cycle intermediates such as succinate, citrate, α-ketoglutarate, and fumarate are important regulators of the production of ROS, and their levels were all decreased extracellularly by SeChry (Figure 3D) [68]. This might suggest that SeChry is also impairing the TCA cycle. Moreover, cysteine synthesis is also affected as CBS is inhibited by SeChry [17], and CBS, besides degrading cysteine, catalyzes cysteine synthesis. Homocysteine originated from the methionine cycle is a direct cysteine precursor [69,70]. The imbalance in amino acid load prompted by SeChry reinforces the role of one-carbon metabolism in cancer cells and demonstrates that its targeting disturbs cell survival.

The serine-glycine one-carbon metabolism integrates inputs from amino acids and glucose derivatives, mainly serine and glycine, producing carbon units (THF4 and derivatives) for redox maintenance and biosynthesis [16]. These carbon units are redistributed via the folate cycle, methionine cycle, and transsulfuration pathway, synthesizing cysteine [71]. Indeed, serine and glycine were found to be impaired by SeChry (Supplementary Figure S3 and Figure 3B,C). Formate was also significantly decreased by SeChry (Figure 3D). In cancer, there is strong evidence for increased mitochondrial formate production [72]. Formate likely contributes to cancer cells’ response to oxidative stress, with the mitochondrial folate pathway generating and releasing excess formate, indicating additional roles in maintaining mitochondrial NADH and NADPH levels [73]. In this context, the decreased levels of serine, glycine, and formate observed after SeChry treatment imply a disruption in cellular redox homeostasis.

Glutamine catabolism is emphasized as crucial in cancer cells, comparable with glucose catabolism, playing an essential role in mitochondrial metabolism [55]. By providing anaplerotic carbons and oxaloacetate, glutamine contributes to the Krebs cycle, supporting ATP production and the synthesis of macromolecules [55,74]. Here, SeChry was observed to significantly decrease intracellular glutamate levels, concomitant with an increase in extracellular glutamine concentrations (Figure 3B,D), indicating that the uptake of glutamine and consequent conversion into glutamate is impaired by SeChry. Thus, as glutaminolysis contributes to the maintenance of the redox state by supporting the synthesis of GSH, these results provide additional support to the notion that SeChry disrupts redox balance, ultimately leading to cell death.

We found that SeChry significantly decreased extracellular pyruvate levels (Figure 3D), potentially due to impaired glucose consumption. This decrease was accompanied by increased levels of branched-chain amino acids (BCAAs; leucine, isoleucine, valine, tyrosine) (Figure 3C). Although mammals do not synthesize BCAAs from pyruvate [75], elevated BCAAs can induce ROS production in PBMCs by activating NADPH oxidase isoforms NOX-1 and NOX-2, and increase mitochondria-derived ROS, leading to oxidative damage and dysfunction [76]. Thus, elevated BCAAs in SeChry-treated cells may exacerbate oxidative stress, contributing to cancer cell death.

As we previously showed that glucose-dependent pathways were pivotal in NSCLC [22], we investigated the potential of inducing an enhanced toxic response by blocking glycolysis with BPA and combining it with SeChry or other chemotherapeutic agents. The combined treatments were more effective in H292 cells (Figure 3F), as they showed the most pronounced differences and with a particularly notable enhancement observed when exposed to BPA, SeChry, and carboplatin. Besides inhibiting glycolysis, BPA acts as an alkylating agent, impairing ATP synthesis by the mitochondrial ETC [77]. This limitation on energy production restricts the growth of many tumors without an apparent effect on non-transformed cells [77]. Given the relatively higher resistance of H292 cells to SeChry compared with A549 and PC-9 cells, the treatment with BPA may disrupt the pathways that allowed cells to adapt to the oxidative stress induced by SeChry. Nevertheless, the combined treatment with SeChry and cisplatin/carboplatin enhanced the cytotoxic response in all the cell lines (Figure 3F–H), suggesting that SeChry would be a good candidate for adjuvant therapy.

Employing lactic acid-conjugated polyurea dendrimer nanoparticles for SeChry delivery, SeChry@PURE_G4_-LA_24_, aimed to enhance the specificity of nanoparticle-targeting to cancer cells, given their generally elevated expression of MCTs. MCTs 1-4 facilitate the crucial transport of compounds like lactate, pyruvate, and ketone bodies across the cell membrane, playing a key role in carbohydrate, fat, and amino acid metabolism [78]. The EC_50_ for SeChry@PURE_G4_-LA_24_ (Figure 4) demonstrated proportionality to the expression levels of MCT1 (Figure 5E). We were not expecting that the human lung bronchial epithelial cell line BEAS-2B would show sensitivity to the nanoparticles, however, this is attributed to the somewhat high expression of MCT1 in these cells. Hence, it is crucial to evaluate these nanoformulations in non-cancer cell lines that do not exhibit elevated levels of both MCT1 and MCT4. BEAS-2B cells were immortalized through adenovirus SV40 transduction, and virus-infected cells commonly exhibit overexpression of MCT1 [79,80] which is related to the increased proliferative capacity of immortalized cells. Nevertheless, the use of MCT1 inhibitors protected BEAS-2B from SeChry@PUREG4-LA24 toxicity and it may be a strategy to protect non-cancer cells in a systemic application of the nanoformulation.

The mechanism by which SeChry@PURE_G4_-LA_24_ enters the cells is still unknown but we speculated that it might occur through a mechanism involving the recognition of MCT transporters. Thus, our goal was to investigate whether inhibiting MCT1 or MCT4 would diminish the sensitivity to SeChry@PURE_G4_-LA_24_. MCT1 inhibitor AZD3965 has shown efficacy as an antitumor agent in different types of cancer, both *in vivo* and *in vitro*, with promising results and a Phase I clinical trial has already been completed in the UK [81]. AZD3965 is a potent and selective MCT1 inhibitor, exhibiting activity against MCT2 while not affecting MCT3/MCT4 [82]. AZD0095, a newer selective inhibitor of MCT4, with a >1000-fold selectivity over MCT1, has not been extensively explored [31]. Our data show that, when comparing to the SeChry@PURE_G4_-LA_24_ single treatment, the double inhibition with AZD3965 plus AZD0095 combined with SeChry@PURE_G4_-LA_24_ further enhanced cell death in PC-9 cells (Figure 5H). However, a small tendency to decrease cell death was observed when SeChry@PURE_G4_-LA_24_ was combined with AZD3965 and AZD0095 in A549 cells and H292 cells (Figure 5F,G). In BEAS-2B the same happened but in a more significant manner, again indicating that these cells also depend on these transporters (Figure 5I). AZD3965 and AZD0095 alone or in combination did not induce cell death in any of the cell lines. While MCT1 inhibition with AZD3965 is demonstrated to increase glycolytic intermediate levels and enzyme activity, causing intracellular lactate accumulation [83], a recent study in human breast cancer cells contradicts this, indicating that AZD3965 does not influence glycolytic activity or intracellular lactate accumulation [40]. Instead, its antitumor effects are attributed to the inhibition of pyruvate export [40]. In human lymphoma and colon carcinoma cells, AZD3965 inhibited both the import and export of monocarboxylates in cells, leading to elevated levels of bioenergy-related metabolites in both MCT4-positive and MCT4-negative cells [84]. Inhibiting MCT1 increased intracellular lactate levels in esophageal adenocarcinoma cells, with a higher increase observed in cells expressing only MCT1 [85]. Indeed, MCT4 expression has been recognized as a resistance mechanism to MCT1 inhibition in tumor cells, and introducing MCT4 into a cell line expressing only MCT1 prevented both lactate accumulation and the inhibition of cell growth by MCT1 inhibitors [86]. While the possibility of these nanoformulations being transported by the MCTs in A549, H292, and BEAS-2B cells is not excluded, the observed changes are not deemed significant. However, targeting MCTs with dendrimers functionalized with lactic acid seems to be a good alternative to specifically deliver drugs to cells overexpressing MCTs. The divergent results in PC-9 cells may be explained by the fact that these cells show both high MCT1 and MCT4 levels [22], and they can have a redundant function. Additionally, a study using MCF7 cells, showed that AZD3965 treatment resulted in the depletion of intracellular GSH, potentially rendering the cells more susceptible to redox-targeted therapy [87]. In this scenario, the concurrent depletion of GSH by both SeChry and AZD3965 in PC-9 cells could enhance a more toxic response. Another explanation could be associated with increased cellular acidosis. Free SeChry was observed to decrease lactate availability in the extracellular media and likely enhance lactate accumulation within the cell (Figure 3D; Supplementary Figures S3 and 5B). Therefore, if the inhibitors are also affecting lactate export, the cytotoxic effect would be intensified. Inhibiting the activity of MCT1 and MCT4, coupled with sustained lactate efflux, induces intracellular acidification, which inhibits LDH activity and ultimately may lead to cell death [23]. Generating MCT1 and/or MCT4 knockout cell lines by CRISPR/Cas9 genome editing technology could be a way to overcome these challenges.

Finally, we also unraveled that SeChry@PURE_G4_-LA_24_ paired with the EGFR inhibitor gefitinib resulted in a greater effect. We found that in all cell lines except for A549, SeChry@PURE_G4_-LA_24_ combined with gefitinib significantly increased cell death when compared with SeChry@PURE_G4_-LA_24_ treatment alone (Figure 5K–P). As mentioned, A549 is a *KRAS*-mutated cell line, and a low response to gefitinib was expected, since in the signaling cascade KRAS is downstream of EGFR, which is the specific target of gefitinib. Accordingly, in the literature, *KRAS-*mutated NSCLC tumors are considered to be poor responders to gefitinib [88]. Interestingly, ROS serve not only as mediators in the EGF/EGFR signaling pathway but also as regulators influencing the oxidation status and activation of EGFR [29]. The oxidation of protein tyrosine phosphatases and specific cysteine residues in EGFR can instigate EGFR-regulated signaling pathways in response to mild ROS levels [89]. Conversely, elevated ROS levels may induce overoxidation of the methionine residue in EGFR^T790M^, leading to the inhibition of the downstream survival pathway of EGFR [28]. This explains the enhanced effect of SeChry and gefitinib since ROS induced by SeChry potentiates EGFR inhibition. Consequently, the modulation of ROS levels in cancer presents a plausible therapeutic strategy for both the treatment and prevention of cancer.

In conclusion, our study not only elucidates the metabolic consequences of SeChry exposure in NSCLC but also introduces a promising nanoformulation strategy for targeted delivery. The observed increased effectiveness with existing therapies underscores the potential of SeChry in enhancing treatment outcomes for NSCLC, opening avenues for future investigations into its broader applicability in cancer therapy.

## Supplementary Material

Supplementary Figures S1-S6

## Data Availability

Data is available in a public repository: https://dev.metabolomicsworkbench.org:22222/data/DRCCMetadata.php?Mode=Study&StudyID=ST003286&Access=QmnZ5992 The DOI for this project (PR002038) is: https://dx.doi.org/10.21228/M85V5Z The study is scheduled to be released on 2024-07-22 (YYYY-MM-DD).

## Abbreviations

ACSS: acetyl-CoA synthetase
CoA: coenzyme A
GLUT: glucose transporter
GPX: glutathione peroxidase
LUAD: lung adenocarcinoma
LUSC: lung squamous cell carcinoma
PC: phosphatidylcholine
PUFA: polyunsaturated fatty acid
SCLY: selenocysteine-lyase
TrxR: thioredoxin reductase
