# Supplementary material for: Metabolic profiling and combined therapeutic strategies unveil the cytotoxic potential of selenium-chrysin (SeChry) in NSCLC cells
Source: Biosci Rep. 2024 Jul 31;44(7):BSR20240752. doi: 10.1042/BSR20240752 (PMC11292474; doi:10.1042/BSR20240752)
Supplement: Supplementary Figures S1-S6 [file BSR-2024-0752_supp.pdf]

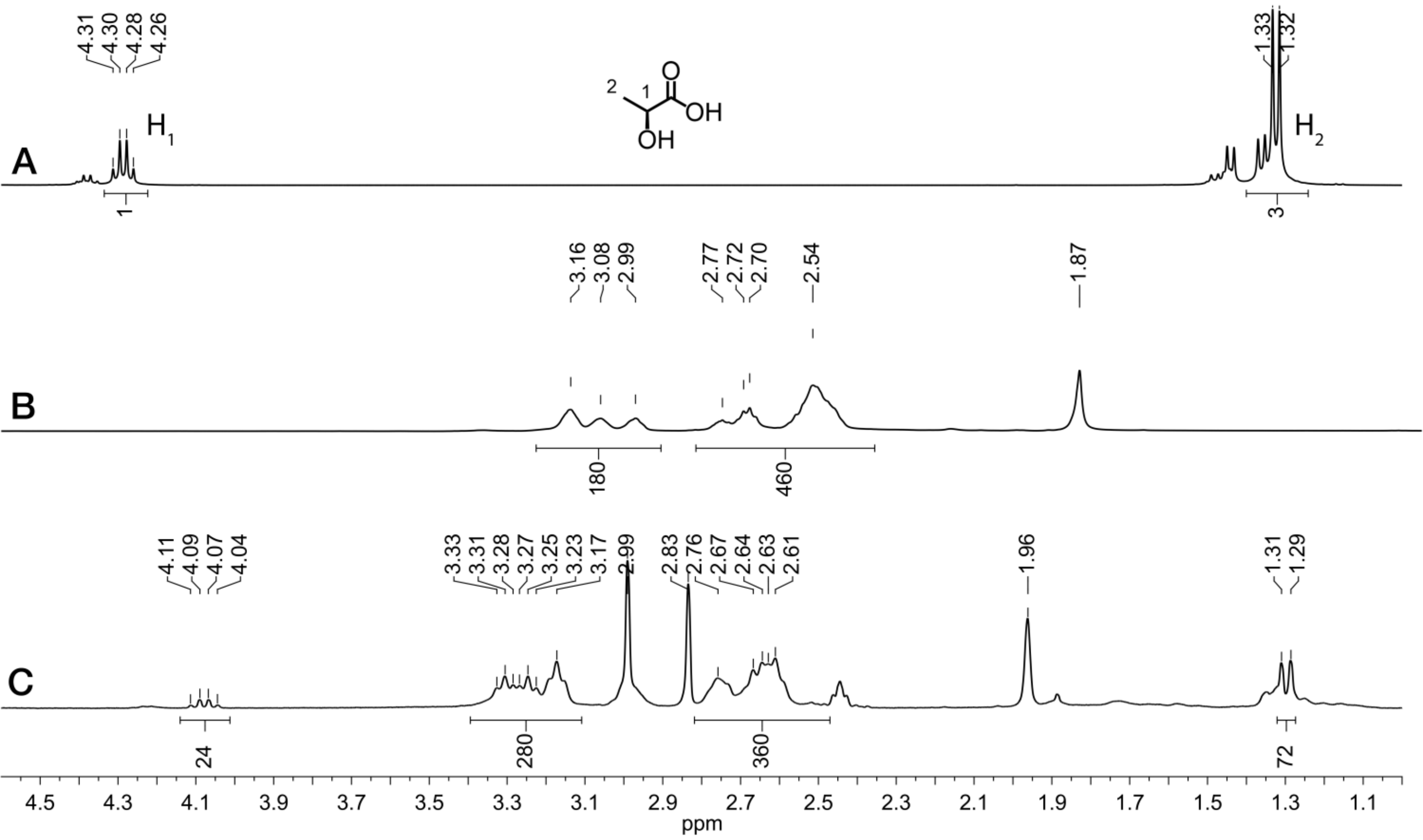

Supplementary Figure 1 – PURE$_{G4}$-LA$_{24}$ dendrimer nanoparticles characterization. ^{1}H-NMR (D_2O) spectra of lactic acid (A), PURE$_{G4}$ (B) and PURE$_{G4}$-LA$_{24}$ (C). ^{1}H NMR (400 MHz, D_2O) δ (ppm): 4.08 (24H, q, *J*= 8.0 Hz, CH lactate), 3.33-3.17 (280H, m, dendrimer), 2.99 (bs, OH lactate), 2.83 (bs, OH lactate), 2.76-2.61 (360H, m, dendrimer), 1.96 (bs, NH dendrimer), 1.30 (72H, d, *J*= 8.0 Hz).

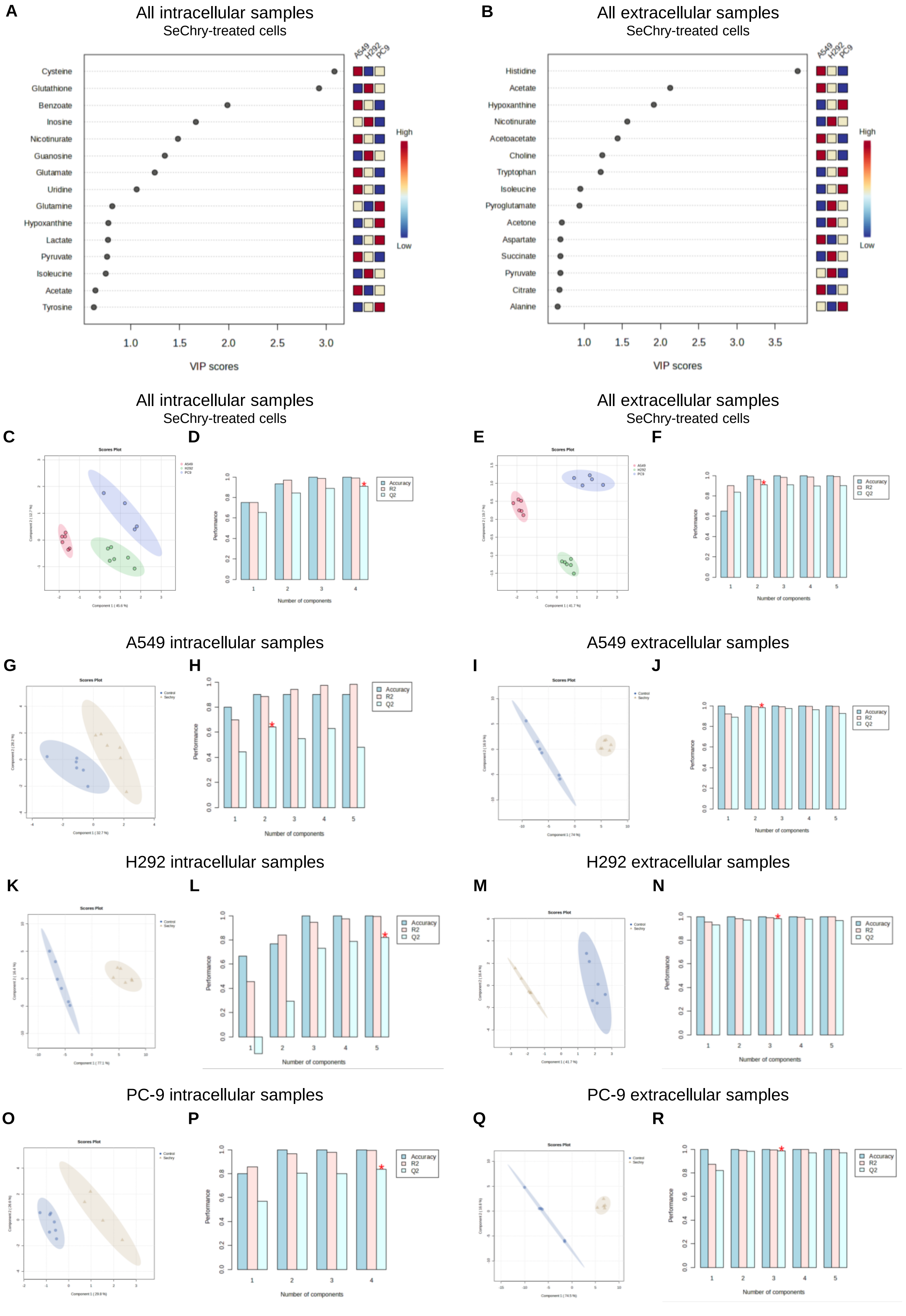

Supplementary Figure 2 – Effect of SeChry on the NSCLC metabolism and most important metabolites contributing to the separation between the control and SeChry-treated cells.

A549, H292 and PC-9 cells were exposed to SeChry for 24 h. Cells were collected, cell extracts were performed, and cell culture media (supernatants) were collected for nuclear magnetic resonance (NMR) spectroscopy analysis. Variable of Importance (VIP) scores plots depicting the 15 most significant metabolites (VIP > 1.0) contributing to the group separations in intracellular (A) and extracellular (B) SeChry-treated cells. Partial Least-Squares Discriminant Analysis (PLS-DA) to infer the influence of SeChry on clustering patterns in (C) cell extracts and (D) supernatant samples of A549, H292 and PC-9. PLS-DA to infer the influence of SeChry on clustering patterns in (G, K, O cell extracts and (I, M, K) supernatant samples of A549, H292 and PC-9, respectively from ^{1}H NMR metabolomic profiles. Values of the PLS-DA classification performance assessed by accuracy, goodness of fit (R2), and predictive ability (Q2) for the top five components in (C) intracellular and (D) extracellular samples of A549, H292 and PC-9. Values of the PLS-DA classification performance in intracellular (H) A549, (L) H292 (P) PC-9 metabolites and in extracellular (J) A549, (N) H292 and (R) PC-9 cells.

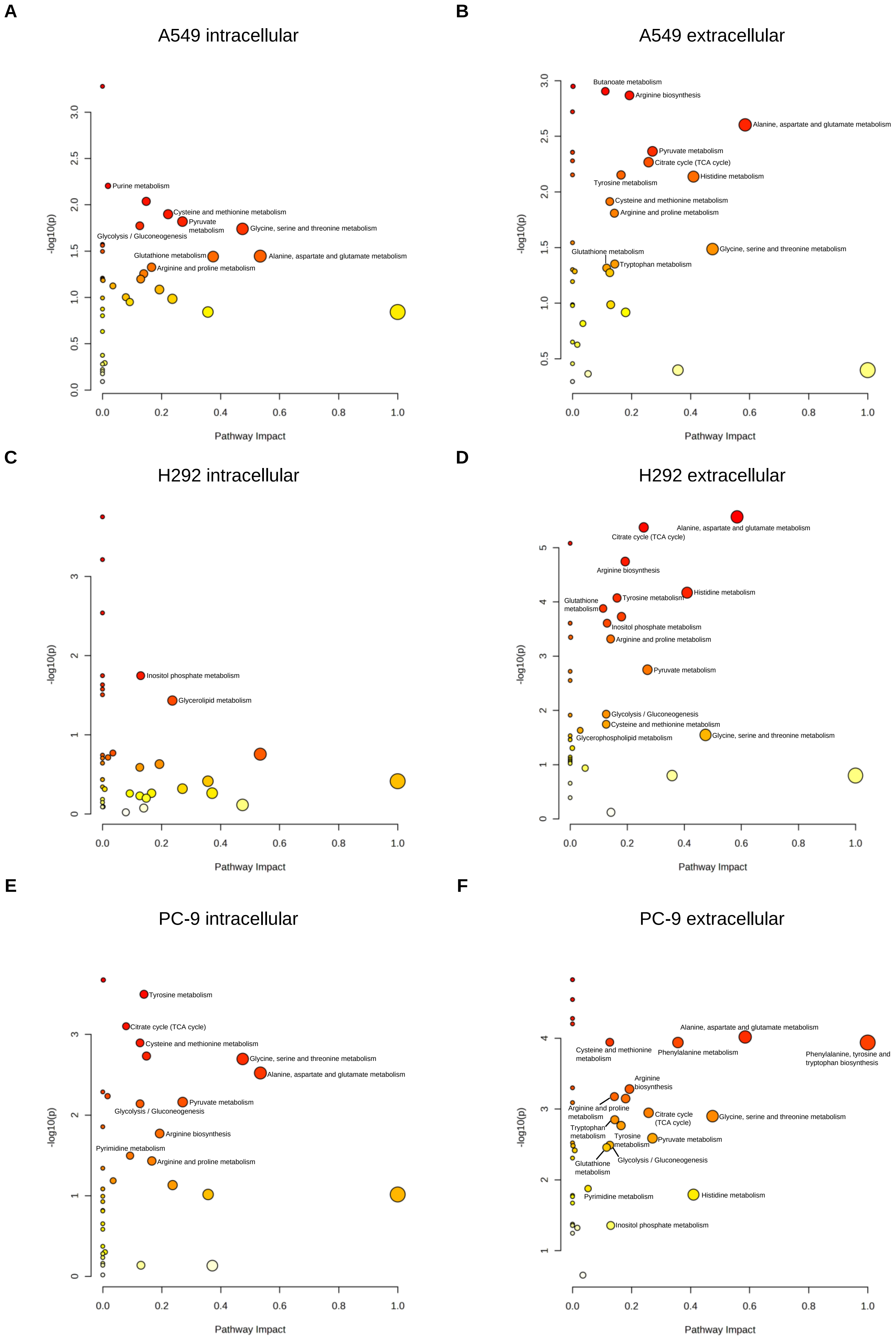

Supplementary Figure 3 - Metabolic pathway enrichment analysis reveals differences in key metabolic pathways across NSCLC cell lines. Metabolic pathway analysis for the effect of SeChry in (A, C, E) intracellular and (B, D, F) extracellular A549, H292 and PC-9 metabolites, respectively. All the matched pathways are displayed as circles. The color and size of each circle are based on the *p*-value and pathway impact value, respectively. The most impacted pathways having statistical significance ($p<0.05$) are indicated. Source: (https://www.metaboanalyst.ca/faces/home.xhtml).

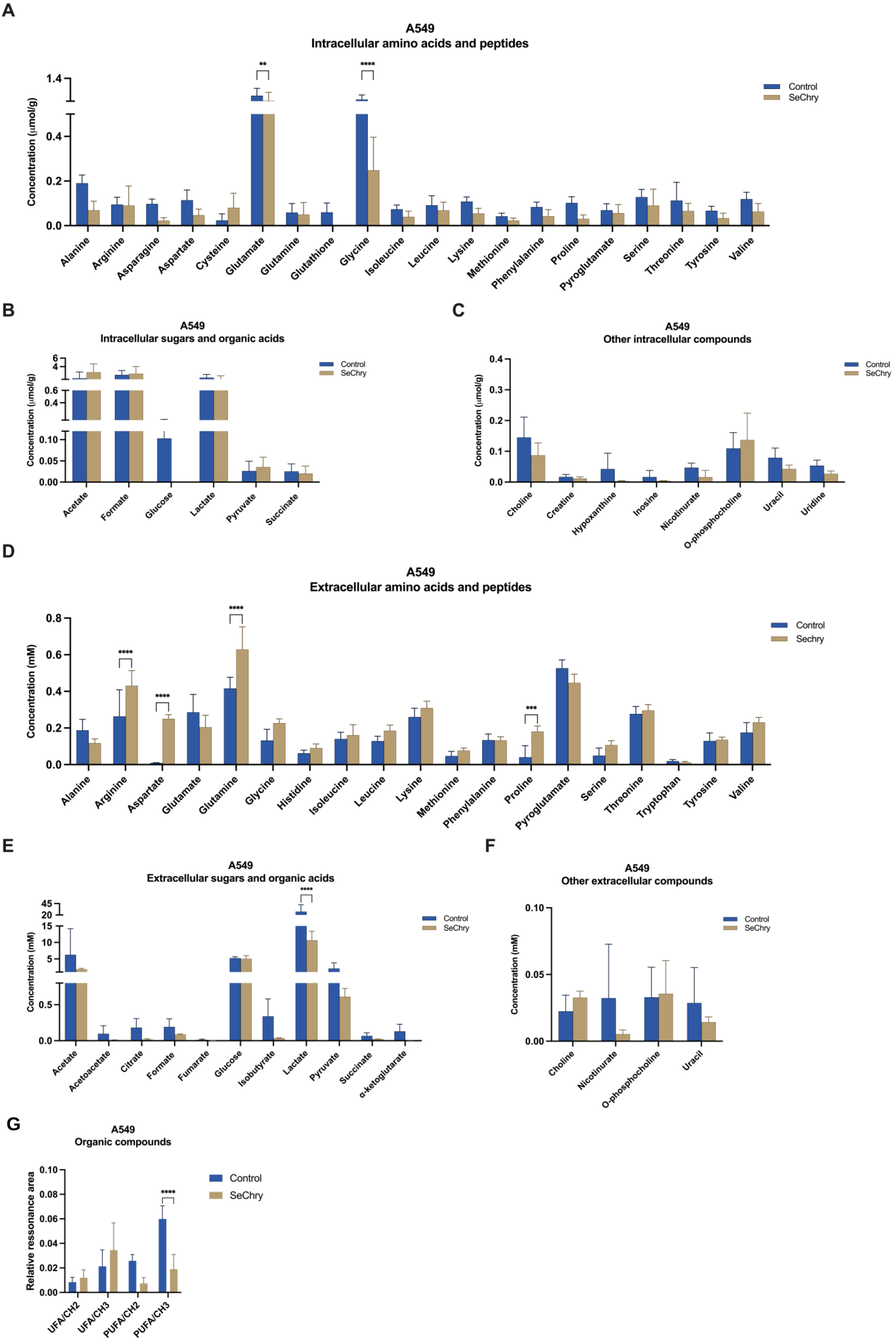

Supplementary Figure 4 – Effect of SeChry on the metabolic profile of A549 cells. A549 cells were exposed to SeChry for 24 h. ^{1}H NMR of A549 cells indicated alterations regarding levels of intracellular (A) amino acids and peptides, (B) sugars and organic acids, and (C) other metabolites and extracellular (D) amino acids and peptides, (E) sugars and organic acids, and (F) other metabolites. (G) Relation between UFA/PUFA and CH2/CH3. UFA, Unsaturated fatty acid; PUFA, Polyunsaturated fatty acid, CH3, methyl group of fatty acids; CH2, methylene group of fatty acids. Data is represented as mean ± SD. *p<0.5, **p<0.01, ***p<0.001, ****p<0.0001 (two-way ANOVA with Tukey's test was used).

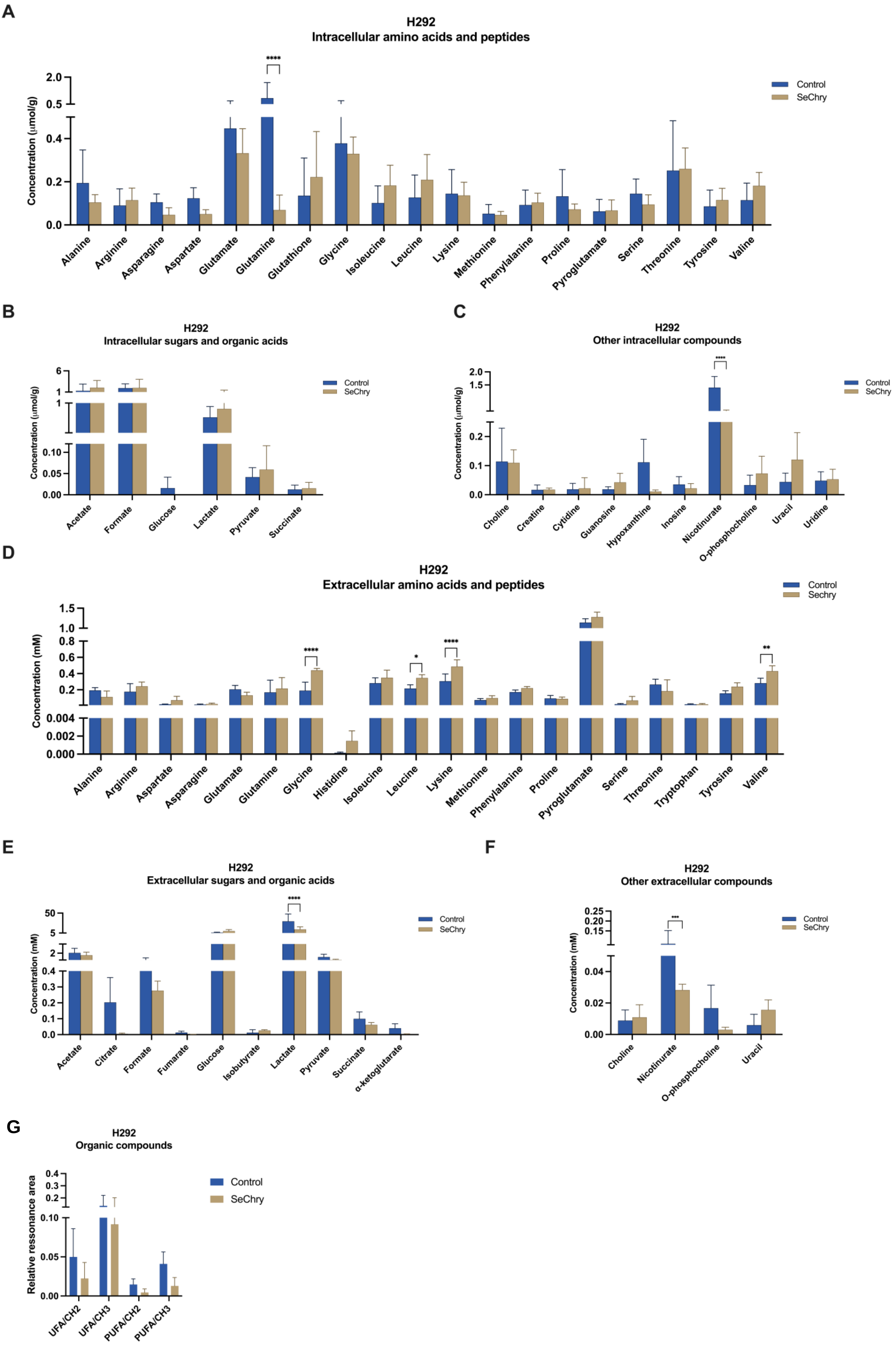

Supplementary Figure 5 - Effect of SeChry on the metabolic profile of H292 cells. H292 cells were exposed to SeChry for 24 h. ^{1}H NMR of H292 cells indicated alterations regarding levels of intracellular (A) amino acids and peptides, (B) sugars and organic acids, and (C) other metabolites and extracellular (D) amino acids and peptides, (E) sugars and organic acids, and (F) other metabolites. (G) Relation between UFA/PUFA and CH2/CH3. UFA, Unsaturated fatty acid; PUFA, Polyunsaturated fatty acid, CH3, methyl group of fatty acids; CH2, methylene group of fatty acids. Data is represented as mean ± SD. *p<0.5, **p<0.01, ***p<0.001, ****p<0.0001 (two-way ANOVA with Tukey's test was used).

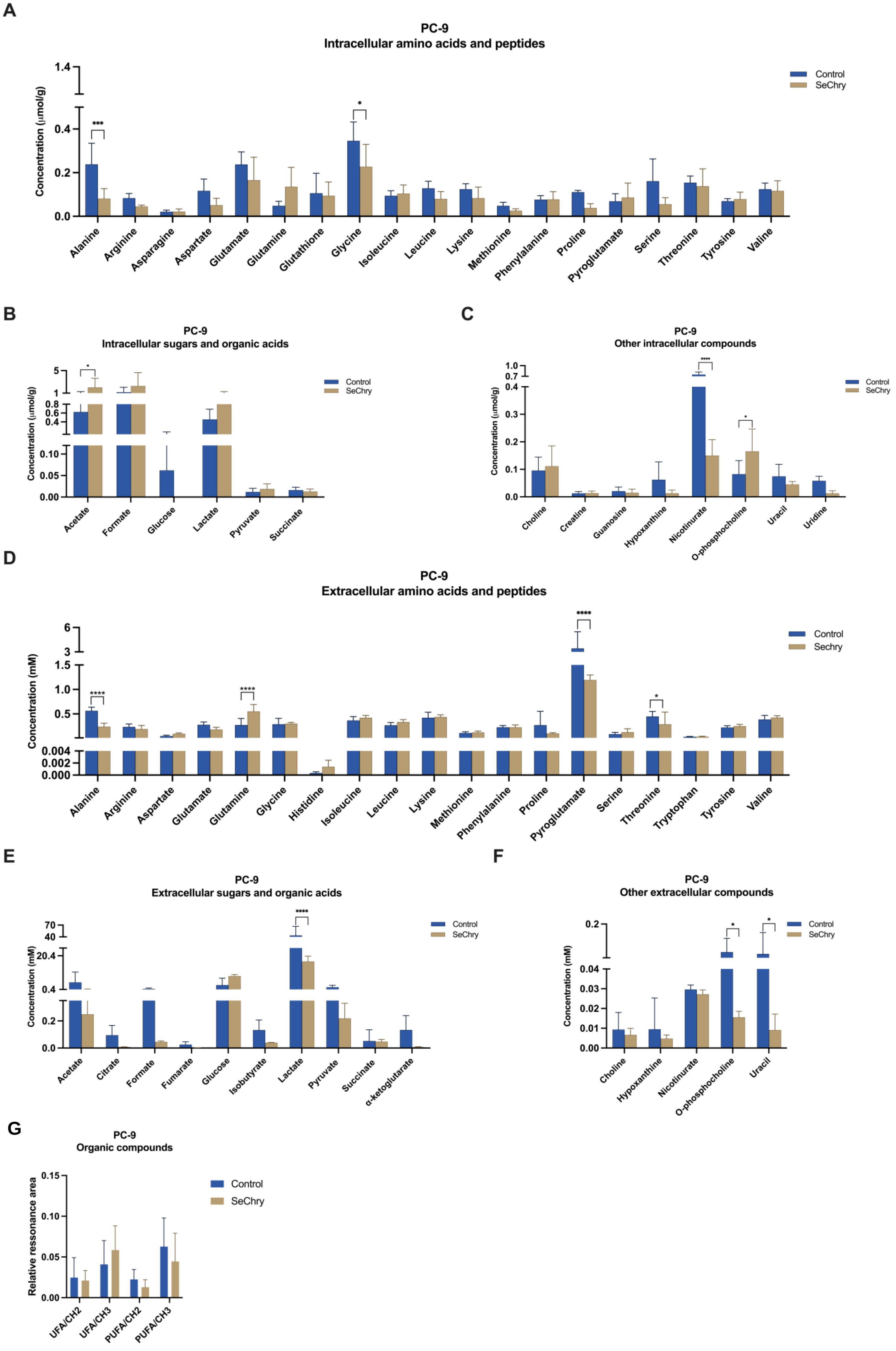

Supplementary Figure 6 - Effect of SeChry on the metabolic profile of PC-9 cells. PC-9 cells were exposed to SeChry for 24 h. ^{1}H NMR of PC-9 cells indicated alterations regarding levels of intracellular (A) amino acids and peptides, (B) sugars and organic acids, and (C) other metabolites and extracellular (D) amino acids and peptides, (E) sugars and organic acids, and (F) other metabolites. (G) Relation between UFA/PUFA and CH2/CH3. UFA, Unsaturated fatty acid; PUFA, Polyunsaturated fatty acid, CH3, methyl group of fatty acids; CH2, methylene group of fatty acids. Data is represented as mean ± SD. *p<0.5, **p<0.01, ***p<0.001, ****p<0.0001 (two-way ANOVA with Tukey's test was used).
